# Achieving one-step molecular photogearing in a minimal light-driven molecular motor

**DOI:** 10.1039/d5sc05065k

**Published:** 2025-09-23

**Authors:** Enrique M. Arpa, Bo Durbeej

**Affiliations:** a Division of Theoretical Chemistry, IFM, Linköping University 58183 Linköping Sweden bodur@ifm.liu.se; b Institute of Organic Chemistry, RWTH Aachen University 52056 Aachen Germany enrique.arpa@rwth-aachen.de

## Abstract

The last decades have seen a wealth of progress in the design and synthesis of molecular motors for converting light energy into directed rotary motion around a double bond. Yet, realizing the full potential of these systems in the field of artificial molecular machines will inevitably require a breakthrough in the formidable challenge to construct molecular photogears for transmitting such motion through space and onto a remote single-bond axis, without losing control of the direction of rotation. Here, we unveil a surprisingly straightfoward mechanism for achieving this goal in a single photochemical step by incorporating a propeller-shaped barrelene motif into the protonated Schiff-base skeleton of a minimal light-driven molecular motor. Corroborating the mechanism by state-of-the-art computational modeling, our study also identifies strategies for optimizing the photogearing efficiency through modulation of steric interactions. Overall, the results of this work help establish a new route for constructing molecular photogears by combining molecular-motor and propeller-shaped structures.

## Abstract 2

This work introduces a new and surprisingly straightforward mechanism for transmitting light-induced rotary motion from a double bond to a single bond in a molecular photogear whose reaction cycle consists of a single photochemical step.

## Graphical abstract

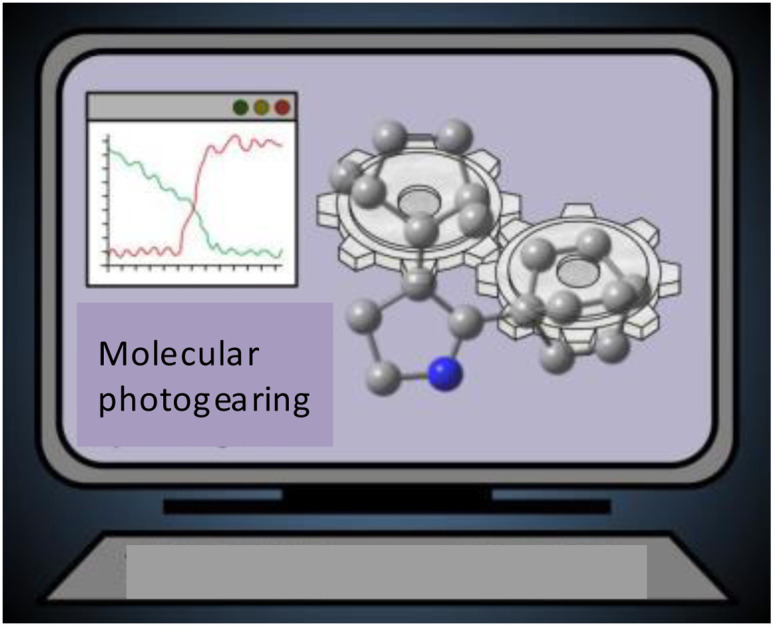

## Introduction

A multitude of machines in our everyday lives are designed in such a way that mechanical motion is transmitted from where it is produced to other components executing the actual function. This paradigm applies also at the nanoscale, as exemplified by Nature-made molecular machines that synthesize ATP (ATP synthase^1–5^) and provide intracellular transport (*e.g.*, kinesin motor proteins).^6–10^ In the field of artificial molecular machines,^11–17^ it is similarly desirable to design and synthesize molecular gears^18–30^ capable of transmitting rotary motion while also controlling the direction of rotation. Here, for the sake of facile motion, constructing gears whose receiver components undergo rotation around a single bond is a natural goal. However, as typical gear designs rely on passive thermal activation, random thermal fluctuations (Brownian motion) make it difficult for them to follow a preferred direction of rotation and, consequently, to perform actual mechanical work.^31^

**Fig. 1:**
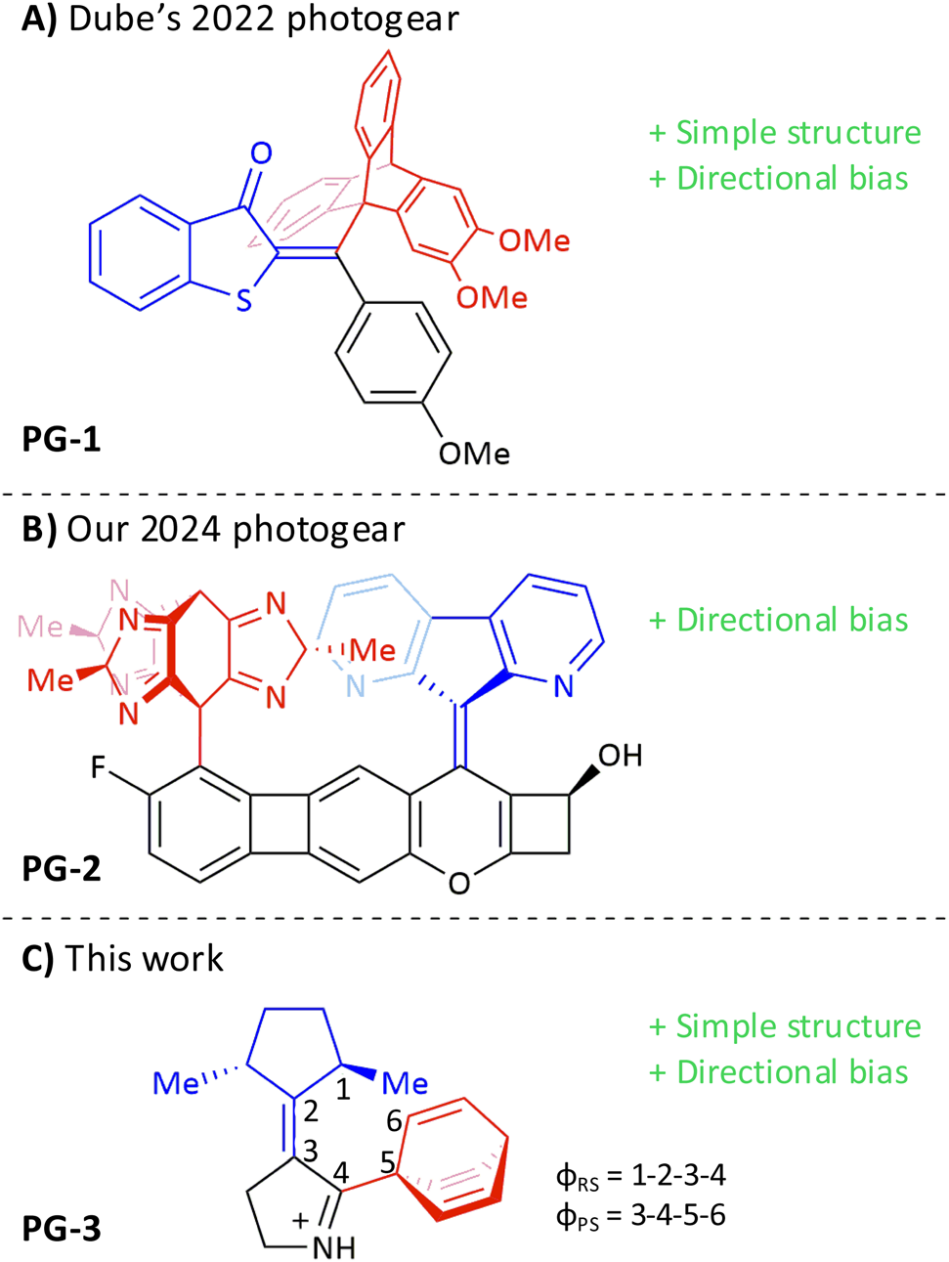
(A) Photogear developed by the Dube group in 2022.^42^ (B) Photogear developed in our 2024 work.^49^ (C) Photogear presented in this work and definitions of relevant dihedral angles. In all photogears, the rotor, propeller and stator parts are blue-, red- and black-colored, respectively.

A possible solution to this problem, advocated by the Feringa group,^32^ is to exploit the unique ability of synthetic molecular motors to convert the energy from an external source into directed rotary motion.^33–41^ Along those lines, in 2022,^42^ the Dube group presented a molecular photogear incorporating a photoswitch of hemithioindigo (HTI) type.^43–45^ Specifically, this design, which is shown as PG-1 in Fig. 1, transmits the photoinduced 180° rotation of the “rotor” moiety of the HTI switch around a C=C bond, into a 120° rotation of a triptycene “propeller” around a C–C bond (in Fig. 1, the rotor and propeller moieties are blue- and red-colored, respectively). Adopting a bevel-like configuration with non-parallel rotation axes, PG-1 differs from previous molecular setups developed by the same group^46,47^ in its ability to transmit the rotary motion through spatial interactions alone (the hallmark of true molecular gearing),^19^ without help from a covalent chain linkage between the rotor and propeller moieties. Following excitation using blue light, the authors showed that PG-1 achieves photogearing with a quantum yield of up to ∼7%, depending on which isomer is irradiated. Moreover, in a follow-up study, the photogearing was found to occur in such a way that both the rotor rotation and the propeller rotation exhibit a rather pronounced directional bias, but no overall directionality.^48^ This likely reflects the absence of a stable and permanent source of chiral asymmetry in PG-1 to control the rotations.

As a potential remedy, we recently put forth the photogear design shown as PG-2 in Fig. 1, whose triptycene-like propeller contains three stereogenic sp^3^ centers.^49^ Combining this propeller with a diazafluorene rotor (fluorene being a common motif in light-driven motors of overcrowded-alkene type),^50,51^ and linking the two *via* an extended “stator” fragment, PG-2 adopts a spur-like configuration with parallel C=C (rotor) and C–C (propeller) rotation axes. Encouragingly, assessing the design by means of quantum chemical calculations and molecular dynamics (MD) simulations, it was found to enable a two-step photogearing cycle with alternating C=C photoisomerization and thermal helix inversion steps.^49^ Furthermore, these steps were predicted to favor directed photogearing both dynamically (during photoisomerization) and kinetically (during thermal helix inversion). However, because of its complex structure, particularly in the stator motif, it would likely be difficult to synthesize PG-2.

Against this background, we herein present and computationally evaluate a new photogear design (PG-3 in Fig. 1) that combines the main favorable attributes of PG-1 (structural simplicity) and PG-2 (directed photogearing) in a reaction cycle involving just one single isomer. In this cycle, photogearing is achieved when the isomer in question reforms itself in a single photochemical step, without any intermediary steps. We refer to such a process as one-step molecular photogearing. Overall, a key goal of our study is to identify the most basic structural ingredients needed to realize molecular photogearing.

## Results and discussion

Our development of PG-3 was guided by a number of considerations. First, we changed from a spur (in PG-2) to a bevel design, for which it is less critical that the rotor-propeller distance is large^24,28,29^ and, consequently, a smaller stator motif might be used. Furthermore, compared to the PG-1 bevel design, the rotor and propeller rotation axes of PG-3 are not directly connected. Second, in order to possibly eliminate thermal steps from the photogearing cycle, the rotor-stator core of PG-3 was based on a protonated Schiff base akin to other such structures computationally predicted (but without rigorous experimental verification) to produce rotary motion for molecular motors in a purely photochemical fashion.^52–55^ Third, as for the choice of propeller, the starting point was again triptycene, but instead of replacing the benzene rings of triptycene with slightly smaller imidazole-tautomeric rings (as done for PG-2), in PG-3 the benzene rings were removed altogether, resulting in a much smaller *C*_3_-symmetric barrelene propeller. Fourth, and finally, instead of having three asymmetric Me groups in the propeller (as in PG-2), in PG-3 two such groups were included in a *C*_2_-symmetric cyclopentane rotor.

**Fig. 2:**
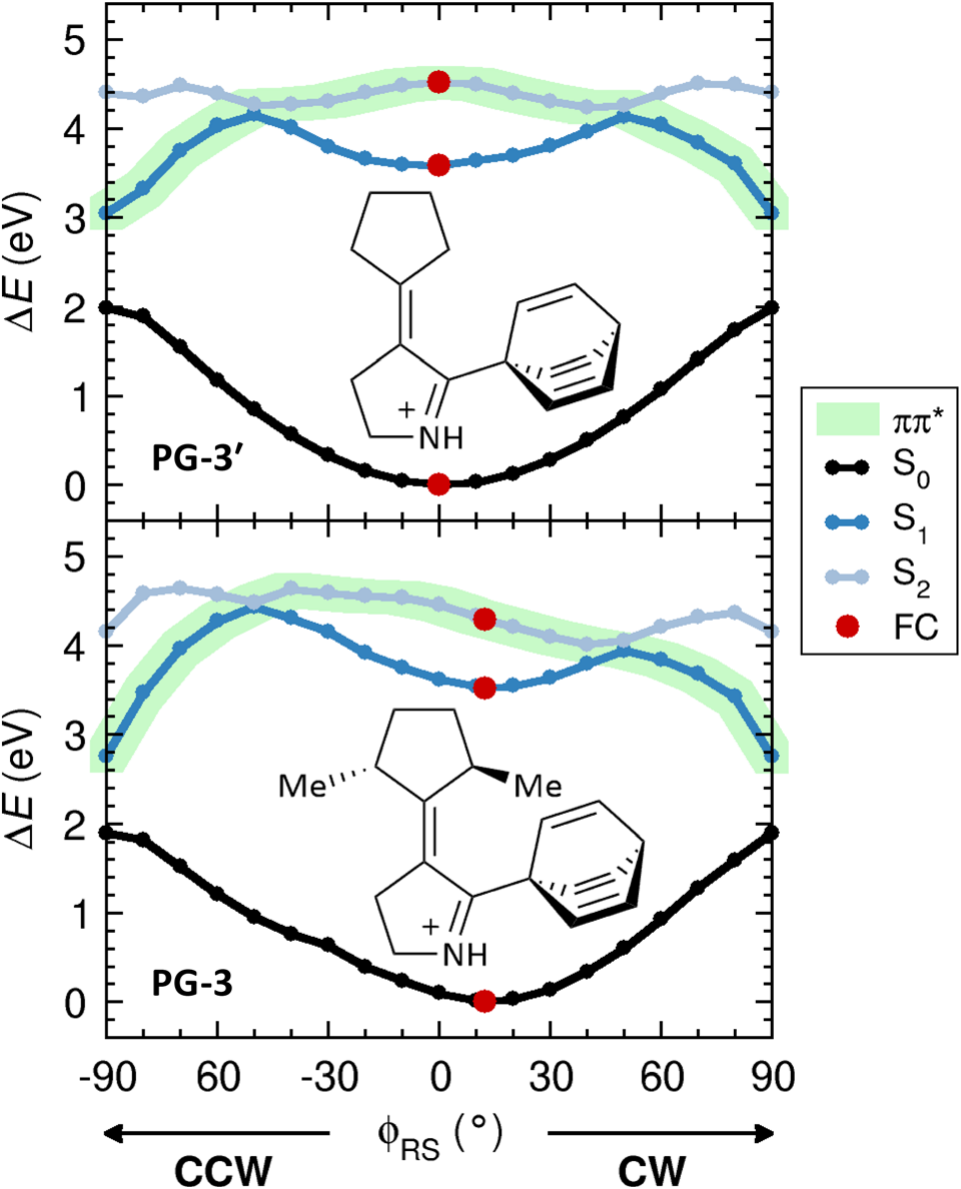
S_0_, S_1_ and S_2_ energy profiles for changing the *φ*_RS_ dihedral angle in the PG-3′ and PG-3 systems, with the ππ* state highlighted in light-green color. In each system, a full red circle indicates the value of *φ*_RS_ at the vertically excited Franck–Condon (FC) point (*i.e.*, at the S_0_ equilibrium geometry).

We began the computational assessment of PG-3 by exploring whether this system has the functionality of a light-driven rotary molecular motor. This requires that the potential energy surface (PES) of the bright ππ* state populated by light absorption exhibits a directional bias for the rotor rotation around the central C=C bond. For PG-3, the idea is that this bias may be realized by the asymmetric rotor Me groups, which introduce steric repulsion between the rotor and the propeller. In order to test this idea, the ππ* PESs of PG-3 and its demethylated derivative PG-3′ (obtained by replacing the Me groups with H atoms) were mapped for both clockwise (CW) and counterclockwise (CCW) changes in the rotor-stator dihedral angle (*φ*_RS_, see Fig. 1C) relative to the value at the ground-state (S_0_) equilibrium geometry. Here, CW/CCW is defined as the direction of rotation in which the value of *φ*_RS_ increases/decreases. Using both density functional theory (DFT) and multiconfigurational methods, the full details of these (and all other) calculations are given in Section 1 of the SI. Including the S_0_ ground and the S_1_ and S_2_ excited states in the analysis, the DFT results are presented in Fig. 2. Very similar results obtained with the multiconfigurational methods are shown and discussed in Section 3 of the SI.

From Fig. 2, it can be seen that the ππ* state of PG-3′ is perfectly symmetric with respect to CW and CCW rotor rotations, which means that this system cannot function as a light-driven rotary motor. PG-3, on the other hand, does display the required directional bias for the rotor rotation. First, the S_0_ equilibrium geometry (which occurs at *φ*_RS_ = 0° for PG-3′) is now markedly twisted in the CW direction (*φ*_RS_ = 11.6°). Second, following excitation to the ππ* state, CW rotation is a downhill process, whereas CCW rotation requires surmounting an energy barrier of 0.34 eV (*ca.* 8 kcal mol^−1^).

Having thus established that PG-3 behaves like a light-driven rotary motor, it should be clarified that the [–90°, 90°] range of *φ*_RS_ values considered in Fig. 2 covers all possible *φ*_RS_ values, thanks to the *C*_2_ symmetry of the rotor. Combined with the *C*_3_ symmetry of the propeller, and the fact that the rotor rotation does not alter the favorable axial orientations of the rotor Me groups, this means that the photochemical rotor rotation should proceed without an accompanying thermal step. This is different from, *e.g.*, overcrowded-alkene motors, where the photochemical steps do change the orientations of the stereogenic substituents from axial to equatorial and, therefore, thermal steps are needed to revert these changes.^41^

**Fig. 3:**
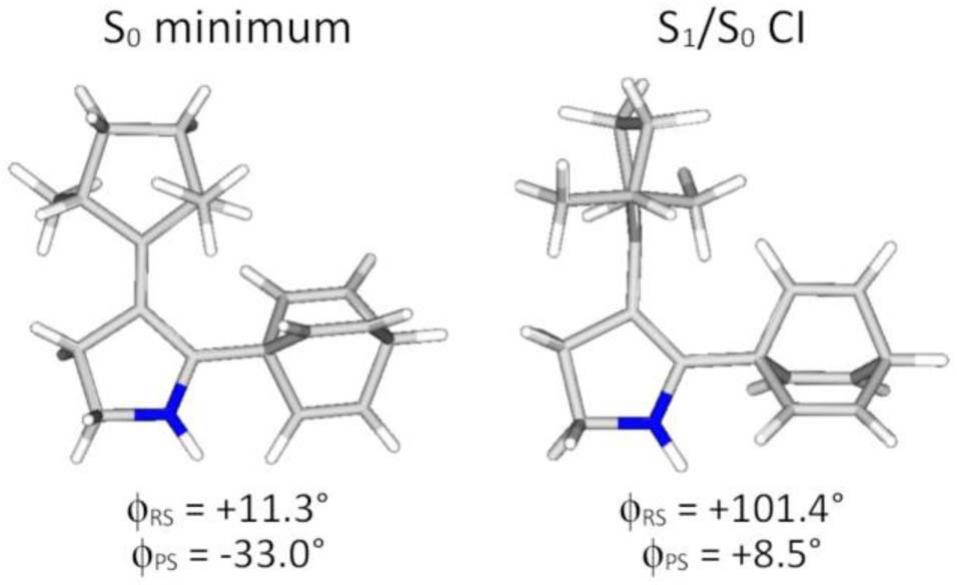
Geometries of the S_0_ minimum and the S_1_/S_0_ CI for rotor rotation for PG-3, and the corresponding values of the *φ*_RS_ and *φ*_PS_ dihedral angles.

Next, we decided to test whether the rotor rotation in PG-3 induces any propeller rotation, as it would if PG-3 also has photogearing capability. To this end, we compared the optimized geometries of the S_0_ minimum of PG-3 and the S_1_/S_0_ conical intersection (CI) through which the rotor rotation of PG-3 was found to proceed. Following typical protocols employed in the literature,^56,57^ these calculations were done with the multiconfigurational complete active space self-consistent field (CASSCF) method.^57,58^ The resulting geometries, alongside the corresponding *φ*_RS_ and propeller-stator (*φ*_PS_, see Fig. 1C) dihedral angles, are shown in Fig. 3.

Interestingly, because of steric clashing between one of the rotor Me groups and the propeller, the ∼90 degree CW rotor rotation needed to reach the S_1_/S_0_ CI causes a ∼42° propeller rotation in the direction of increasing *φ*_PS_ values, which we define as CCW. This suggests that PG-3 could well be capable of photogearing. Furthermore, since the rotary cycle underlying the basic motor function of PG-3 appears to require only one single photoinduced 180° rotor rotation to regenerate the parent S_0_ minimum (thanks to the rotor and propeller symmetries), it seems possible that any photogearing by PG-3 would also proceed through a “simple” mechanism. On the downside, the high symmetry of PG-3 would inevitably make experimental observation of the photogearing more difficult.^48^

In order to model the actual photodynamics of PG-3 upon light absorption and assess the propensity of this compound to sustain photogearing in as unbiased fashion as possible, we next decided to perform non-adiabatic molecular dynamics (NAMD) simulations.^59,60^ Continuing to use the CASSCF method and running the simulations for 500 fs, the details of the simulations are provided in Section 1 of the SI. In principle, following excitation to the ππ* state of PG-3, four different types of outcomes are possible and uniquely characterizable in terms of the changes Δ*φ*_RS_/Δ*φ*_PS_ in *φ*_RS_/*φ*_PS_ dihedral angles along the NAMD trajectories. Specifically, with the above definitions of CW and CCW directions of rotations for the rotor and the propeller, respectively, a positive Δ*φ*_RS_ value (*i.e.*, an increase in *φ*_RS_) corresponds to a CW rotor rotation, whereas a positive Δ*φ*_PS_ value (*i.e.*, an increase in *φ*_PS_) corresponds to a CCW propeller rotation. Thereby, the different outcomes can be described as follows:

• Photogearing: this is when a CW rotor rotation (Δ*φ*_RS_ = +180°) induces a CCW propeller rotation (Δ*φ*_PS_ = +120°), or a CCW rotor rotation (Δ*φ*_RS_ = −180°) induces a CW propeller rotation (Δ*φ*_PS_ = −120°). Here, we refer to the first scenario as forward photogearing, and the second scenario as reverse photogearing. Notably, the results from the static calculations in Fig. 2 and 3 suggest that forward photogearing is more likely than reverse photogearing.

• Slippage: this is a free-standing CW or CCW rotor rotation (Δ*φ*_RS_ = ±180°) that occurs without a propeller rotation (Δ*φ*_PS_ = 0°), or a free-standing CW or CCW propeller rotation (Δ*φ*_PS_ = ±120°) that occurs without a rotor rotation (Δ*φ*_RS_ = 0°).

• Gear clash: this is when a CW rotor rotation (Δ*φ*_RS_ = +180°) induces a CW propeller rotation (Δ*φ*_PS_ = −120°), or a CCW rotor rotation (Δ*φ*_RS_ = −180°) induces a CCW propeller rotation (Δ*φ*_PS_ = +120°). As appreciable rotor-propeller steric repulsion would have to be overcome for gear clashing to happen, this outcome seems unlikely.

• FC repopulation: this is when the rotor begins rotating in one direction, reaches the associated S_1_/S_0_ CI, decays to the S_0_ state, but then continues rotating back in the opposite direction to repopulate the parent S_0_ minimum (Δ*φ*_RS_ = 0° and Δ*φ*_PS_ = 0°).

**Fig. 4:**
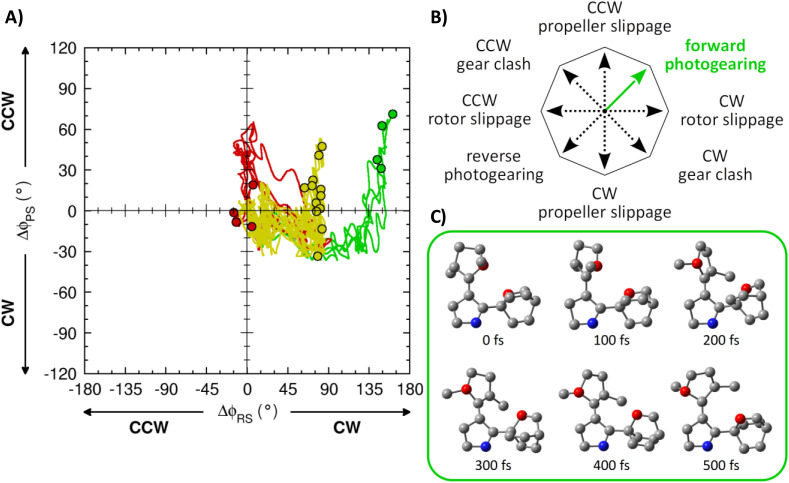
(A) Key structural changes along the 20 NAMD trajectories calculated for PG-3. The color-coding used for plotting the trajectories is explained in the main text. For ease of interpretation, the final structure of each trajectory is encircled. The plot shows the changes in the *φ*_RS_ and *φ*_PS_ dihedral angles (Δ*φ*_RS_ and Δ*φ*_PS_) relative to the unique *φ*_RS_ and *φ*_PS_ values at the starting point of each trajectory. Accordingly, each trajectory begins at Δ*φ*_RS_ = 0° and Δ*φ*_PS_ = 0°. (B) Schematic illustration of all possible reaction outcomes (except FC repopulation) following photoexcitation of PG-3. (C) Structural snapshots at 100 fs intervals from a trajectory showing forward photogearing, with all hydrogen atoms hidden and two carbon atoms highlighted in red color for ease of interpretation. Notice how the positions of the highlighted carbon atoms gradually change from right to left (the rotor carbon), and from back to front (the propeller carbon), during the CW rotor and CCW propeller rotations.

The calculated NAMD trajectories are summarized in Fig. 4, which shows all possible Δ*φ*_RS_ and Δ*φ*_PS_ values among the 20 trajectories at each time step. An alternative way of presenting these results is through Fig. S6 of the SI, which plots the Δ*φ*_RS_ and Δ*φ*_PS_ values for the individual trajectories separately. Before commenting on the results, it should be noted that the purpose of the NAMD simulations is not to obtain a quantitative estimate of the photogearing quantum yield of PG-3 – this would require calculating many more and much longer trajectories, which is not yet affordable for CASSCF-based NAMD simulations of systems the size of PG-3 (44 atoms). Rather, the purpose is to probe, qualitatively, whether photogearing is a viable reaction channel. Besides emphasizing this point, it may also be noted that the necessity to limit the number of states included in the simulations (2, S_0_ and S_1_) implies that channels involving other excited states than the bright ππ* state will not be manifested.

Finding that all of the 20 trajectories in Fig. 4 can be classified into one of three different categories, shown in green, red and yellow colors, it is notable that four of them (20%, green color) induce forward photogearing and decay to the S_0_ state before the 500 fs mark. In fact, the number of such trajectories is identical to the number of trajectories leading to FC repopulation (20%, red color). Combined with the fact that the remaining 12 trajectories (60%, yellow color) show no indication that other processes would outcompete photogearing, these results lend credence to the idea that photogearing is indeed a feasible reaction channel (the 12 trajectories in question reach the S_1_/S_0_ CI region but do not decay to the S_0_ state within 500 fs). At the same time, despite the absence of any imposed reaction coordinate in the NAMD simulations, it is certainly possible for the simulations to be biased toward forward photogearing, in the same way as previous studies have documented bias in the predicted outcomes of other photochemical reactions by computational modeling.^61^

Nonetheless, as further evidence that the four green-colored trajectories in Fig. 4 represent forward photogearing, propagating them adiabatically in the S_0_ state for an additional 500 fs readily produced a full CCW propeller rotation with Δ*φ*_PS_ = +120° (see Fig. S7 and Section 5 of the SI, as well as the multimedia file described in Section 7 of the SI). Analogous extended simulations were also performed for the four red-colored trajectories in Fig. 4 (see Section 5 of the SI).

Based on the results in Fig. 4, two additional comments are in place. First, we note that photogearing occurs exclusively in the forward direction, whereby a CW rotor rotation induces a CCW propeller rotation. This finding is consistent with the prediction of a preferred photogearing direction by the static calculations in Fig. 2 and 3. Second, we note that the photogearing is asynchronous, with the propeller rotation lagging behind the rotor rotation quite considerably. In fact, although the propeller rotation is set in motion in the S_1_ state (as can be inferred from Fig. 3), most of the rotation occurs when the photoexcited system has decayed to the S_0_ state through the CI for rotor rotation, and quite large Δ*φ*_RS_ values have been attained (see Fig. 4).

Importantly, maximally efficient photogearing also requires that neither rotor rotation nor propeller rotation can be triggered thermally. Accordingly, it is advantageous if the thermal barriers for free-standing rotor and propeller rotations are large. Hence, we calculated the barriers of these “slippage” processes for PG-3 with two different density functionals, as shown in Table S2 of the SI. From these calculations, it can be seen that the free-energy barrier for rotor slippage around the associated double bond is of such magnitude (33–40 kcal mol^−1^) that this process does not influence the photogearing efficiency. The barrier for single-bond propeller slippage, on the other hand, is sufficiently small (only 7–8 kcal mol^−1^) that this moiety is likely to start rotating in the dark, which would be detrimental to photogearing. However, this appears to be a manageable issue, because complementary calculations also included in Table S2 identified two different strategies for raising this barrier within the confines of the PG-3 template. More specifically, these strategies are represented by the PG-3 derivatives shown in Fig. S9 of the SI.

The first strategy is to increase the steric repulsion between the propeller and the stator, which can be done by replacing the protonated Schiff base of the stator with a methylated ditto. This was found to raise the barrier to 10–11 kcal mol^−1^ (see Table S2). The second strategy is to instead increase the steric repulsion between the propeller and the rotor, which is readily achieved by replacing the small barrelene propeller with the larger triptycene propeller so commonly employed by thermally driven molecular gears^24,28,29^ (and also by the PG-1 photogear in Fig. 1).^42^ Encouragingly, this raised the barrier to a considerable 20–23 kcal mol^−1^ (see Table S2).

Here, it should be pointed out that the indirect, positive effect on the photogearing efficiency that these two strategies offer through inhibition of propeller slippage, could be offset by a direct, negative effect from the altered steric interactions they introduce. While an analysis of this possibility is beyond the scope of the present work (and will have to await future NAMD simulations of the larger PG-3 derivatives in Fig. S9), it deserves to be mentioned that the electronic structure of the rotor-stator core of PG-3 was found to be quite inert to the altered steric interactions. This may be interpreted to indicate that any such negative effect is not pronounced.

Finally, it is clear that synthetic feasibility would matter for experimental realization and testing of the PG-3 design. Pleasingly, synthetic procedures are available for both the rotor-stator core^54,62^ and the barrelene propeller.^63–65^ Still, barrelenes are sometimes regarded as “synthetically challenging”.^65^ Furthermore, barrelenes can photoisomerize into cyclooctatetraenes and semibullvalenes,^66^ which also would have to be dealt with. Again, as was the case for propeller slippage, a viable solution to these challenges could be to replace barrelene with triptycene (see Fig. S9). Indeed, the synthesis of triptycenes is well developed.^67,68^ Moreover, their larger size offers further opportunities to reducing the propeller symmetry through chemical modification, which would make it easier to observe the photogearing experimentally.

## Conclusions

In summary, we have discovered a simple mechanism for realizing molecular photogearing in a compound where a *C*_3_-symmetric propeller is added to a minimal light-driven molecular motor of protonated Schiff-bass type.^52–55^ Specifically, we have shown that combining these building blocks produces a photogear whose through-space transmission of double-bond rotary motion into directed single-bond rotary motion occurs in a single step, and whose efficiency is tunable through steric interactions. Altogether, the results of this work identify a path toward progress on a problem of profound importance in the field of artificial molecular machines.

## Author contributions

The authors contributed equally to all parts of this work.

## Conflicts of interest

There are no conflicts to declare.

## Supplementary Material

SC-OLF-D5SC05065K-s001

SC-OLF-D5SC05065K-s002

## Data Availability

The data supporting this article have been included as part of the SI. Supplementary information: computational details, additional results (Fig. S1–S9 and Tables S1 and S2), description of SI multimedia file, and Cartesian coordinates and energies of optimized geometries. See DOI: https://doi.org/10.1039/d5sc05065k.
