# Supplementary material for: Achieving one-step molecular photogearing in a minimal light-driven molecular motor
Source: Chem Sci. 2025 Sep 23;16(42):19910–6. doi: 10.1039/d5sc05065k (PMC12477632; doi:10.1039/d5sc05065k)
Supplement: SC-016-D5SC05065K-s001 [file SC-016-D5SC05065K-s001.pdf]

Supplementary Information (SI) for Chemical Science.
This journal is © The Royal Society of Chemistry 2025

Electronic supplementary information

Achieving one-step molecular photogearing in a minimal light-driven molecular motor

Enrique M. Arpa*[ab] and Bo Durbeej*[a]

Table of contents

1. Computational details Page S1
2. Vertical excitation energies Page S2
3. Comparative rotor-stator dihedral scans Page S3
4. Size of the active space Page S4
5. MD simulations Page S5
6. Thermal barriers for rotor and propeller slippage processes Page S8
7. Description of supplementary multimedia file Page S9
8. References Page S10
9. Cartesian coordinates and energies of optimized geometries Page S11

---

[a] Division of Theoretical Chemistry, IFM
Linköping University
58183 Linköping
Sweden
E-mail: bodur@ifm.liu.se

[b] Institute of Organic Chemistry
RWTH Aachen University
52056 Aachen
Germany
E-mail: enrique.arpa@rwth-aachen.de

1. Computational details

Vertical excitation energies. Vertical excitation energies of **PG-3** were calculated in the following way. First, the S_0 geometry of **PG-3** was optimized at the DFT level, using the CAM-B3LYP exchange-correlation functional[1] and the cc-pVDZ basis set[2] (through a frequency calculation at the same level, the resulting geometry was found to be a potential-energy minimum with real vibrational frequencies only). Based on this geometry, vertical excitation energies and the corresponding oscillator strengths were then calculated with both CAM-B3LYP (in the framework of time-dependent DFT (TD-DFT)[3]) and the XMS-CASPT2 multiconfigurational second-order perturbation theory method[4–6] in combination with the larger cc-pVTZ basis set.[2] These, and all other DFT and multiconfigurational calculations, were done with the Gaussian 16[7] and OpenMolcas 23.10 software packages,[8] respectively.

The XMS-CASPT2 calculations made use of an active space comprising four electrons distributed in the four orbitals shown in Fig. S1. Following benchmark tests described in Section 4 of this document, this (4,4) active space, without any orbitals located at the barrelene propeller, was employed for all multiconfigurational calculations reported in the present work except where otherwise noted. The reference CASSCF wave function for the S_1 state was computed in a state-averaged fashion, including the S_0 and S_1 states with equal weights (SA2-CASSCF). The calculated excitation energies are presented in Section 2 of this document.

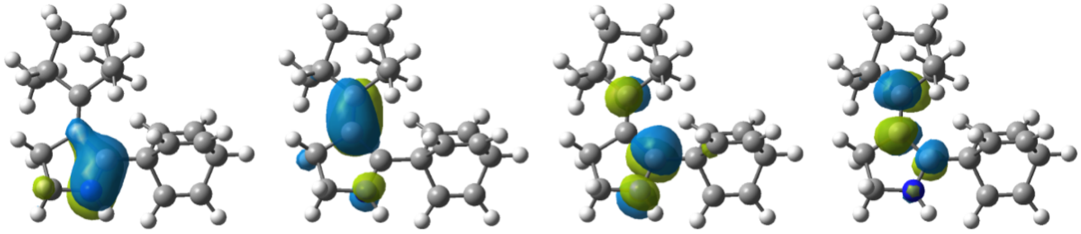

Fig. S1 CASSCF/cc-pVTZ active-space orbitals in the S_1 Franck-Condon (FC) region of **PG-3**, with occupation numbers given in parentheses.

The reason for using a small (4,4) active space for the multiconfigurational calculations is two-fold. First, this ensures that the bright and photochemically relevant ππ* state of **PG-3** is the lowest singlet excited state (i.e., S_1), which simplifies the CASSCF-based NAMD simulations by making it possible to only consider the S_0 and S_1 states. Indeed, with a larger active space that also includes orbitals located at the propeller, the S_1 state becomes a dark charge-transfer (CT) state formed upon excitation from a π orbital of the propeller to a π* orbital of the rotor-stator Schiff-base core. Second, from the viewpoint of computational cost, the use of a small active space is an absolute necessity for CASSCF-based NAMD simulations of systems the size of **PG-3** (44 atoms). As a validation of the (4,4) active space, Section 4 of this document presents results from comparative multiconfigurational calculations performed with a larger active space.

Rotor-stator dihedral scans. The rotor-stator dihedral scans in Fig. 2 of the main text and Section 3 of this document were calculated in the following way. First, using CAM-B3LYP/cc-pVDZ, constrained S_0 geometry optimizations were carried out in which the ϕ_{RS} dihedral angle of the two systems in question (**PG-3** and its demethylated derivative **PG-3'**) was varied in steps of 10° between –90° and +90°. Throughout all optimizations, all geometric coordinates except ϕ_{RS} were relaxed. Based on the resulting geometries, the rotor-stator dihedral scans were then obtained by calculating vertical excitation energies at the TD-CAM-B3LYP/cc-pVTZ (**PG-3** and **PG-3'**), SA2-CASSCF/cc-pVTZ (**PG-3**) and XMS-CASPT2/cc-pVTZ (**PG-3**) levels of theory.

CASSCF geometry optimizations. The geometries of the S_0 minimum of **PG-3** and the associated S_1/S_0 CI for rotor rotation shown in Fig. 3 of the main text were optimized at the SA2-CASSCF/cc-pVDZ level of theory. As appropriate, through a frequency calculation at the same level, the S_0 minimum was found to have real vibrational frequencies only.

NAMD simulations. The NAMD simulations of **PG-3** were carried out with Tully's fewest-switches surface-hopping algorithm,[9] as implemented in OpenMolcas 23.10.[8] Full details of this implementation are available elsewhere.[8,10] Including the S_0 and S_1 states in the simulations and starting them in the S_1 state with 20 different initial nuclear configurations and velocities, the trajectories were calculated at the SA2-CASSCF/6-31G(d) level of theory, using an integration time step of 40 a.u. (~1 fs) for the classical propagation of the nuclei. The initial conditions were generated from a harmonic-oscillator Wigner distribution of S_0 vibrational frequencies calculated at the same level. While the simulations were run for 500 fs, following decay to the S_0 state some of the trajectories were propagated adiabatically (i.e., without surface hopping) in this state for an additional 500 fs, as further discussed in Section 5 of this document.

Thermal barriers for rotor and propeller slippage processes. The thermal barriers for rotor and propeller slippage processes discussed in Section 6 of this document were calculated using two different density functionals, CAM-B3LYP and M06-2X[11] (the latter of which may be particularly well-suited for the description of weak dispersion interactions[11]). First, the associated transition structures were optimized at the CAM-B3LYP/cc-pVDZ and M06-2X/cc-pVDZ levels of theory. For each transition structure obtained, a frequency calculation (carried out at the same level as the preceding geometry optimization) was then performed to confirm the presence of one imaginary vibrational frequency along the normal mode of interest, as well as to obtain Gibbs free energies at room temperature. The chemical relevance of each transition structure was corroborated by means of intrinsic reaction coordinate calculations.[12] Finally, more accurate electronic energies of the transition structures were obtained through single-point calculations with the cc-pVTZ basis set.

2. Vertical excitation energies

The vertical excitation energies calculated for **PG-3** (and, for comparison, **PG-3'**) are given in Table S1. The orbitals involved in the excitations are shown in Fig. S1 (XMS-CASPT2 calculations) and S2 (TD-CAM-B3LYP calculations). At the TD-CAM-B3LYP level, the bright $\pi\pi^*$ state corresponds to S_2 and has an energy of 4.29 eV. S_1, in turn, has an energy of 3.52 eV and is a dark CT state formed upon excitation from a π orbital of the propeller to a π^* orbital of the rotor-stator Schiff-base core. Notably, with the active space for the XMS-CASPT2 calculations selected in such a way that it does not include any orbitals located at the propeller (see Fig. S1), this CT state is (by design) not found by XMS-CASPT2. Thus, at this level, the bright $\pi\pi^*$ state is instead S_1. Pleasingly, the corresponding energy (3.92 eV) is similar to that predicted by TD-CAM-B3LYP (4.29 eV).

Table S1 Vertical excitation energies (ΔE) of **PG-3** and (for comparison) **PG-3′** calculated at the TD-CAM-B3LYP and XMS-CASPT2 levels of theory

	**PG-3** TD-CAM-B3LYP	**PG-3** TD-CAM-B3LYP	**PG-3** XMS-CASPT2	**PG-3′** TD-CAM-B3LYP	**PG-3′** TD-CAM-B3LYP
Excitation	$S_0 \rightarrow S_1$	$S_0 \rightarrow S_2$	$S_0 \rightarrow S_1$	$S_0 \rightarrow S_1$	$S_0 \rightarrow S_2$
ΔE [a]	3.52 (353)	4.29 (289)	3.92 (316)	3.59 (345)	4.52 (275)
f [b]	<0.001	0.282	0.390	0.001	0.346
Orbitals involved [c] (contribution)	$\pi_{1b} \rightarrow \pi_{1b}^*$ (99%)	$\pi_{2b} \rightarrow \pi_{1b}^*$ (96%)	$\pi_{2a} \rightarrow \pi_{1a}^*$ (95%)	$\pi_{1b} \rightarrow \pi_{1b}^*$ (99%)	$\pi_{2b} \rightarrow \pi_{1b}^*$ (98%)

[a] Energies are given in eV and (in parentheses) nm. [b] Oscillator strengths. [c] The orbitals involved in the excitations are depicted in Fig. S1 (XMS-CASPT2) and S2 (TD-CAM-B3LYP).

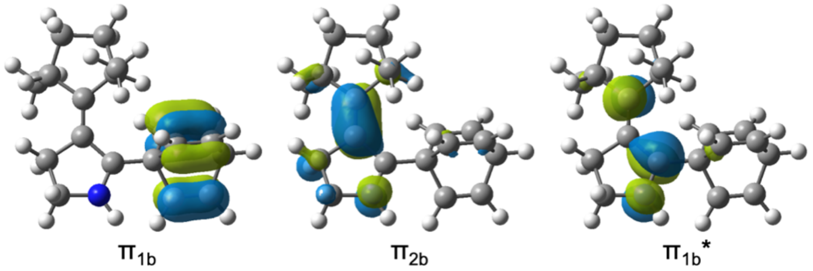

Fig. S2 CAM-B3LYP/cc-pVTZ orbitals involved in the $S_0 \rightarrow S_1$ and $S_0 \rightarrow S_2$ excitations of **PG-3**. The orbitals involved in the corresponding excitations of **PG-3′** are almost identical, and are therefore not shown.

3. Comparative rotor-stator dihedral scans

While the rotor-stator dihedral scans in Fig. 2 of the main text were calculated at the DFT level, the NAMD simulations in Fig. 4 were performed at the SA2-CASSCF level. Accordingly, it is of interest to ascertain that these two levels yield consistent and accurate results. To this end, for **PG-3**, the original scan in Fig. 2 obtained by TD-CAM-B3LYP single-point calculations was compared with the corresponding scans produced by both SA2-CASSCF and XMS-CASPT2 single-point calculations. This is done in Fig. S3. Encouragingly, the three levels are found to yield almost identically shaped energy profiles favoring a barrierless CW rotor rotation and showing energy barriers for the CCW rotor rotation that are very similar (0.34, 0.39 and 0.45 eV for TD-CAM-B3LYP, XMS-CASPT2 and SA-CASSCF, respectively).

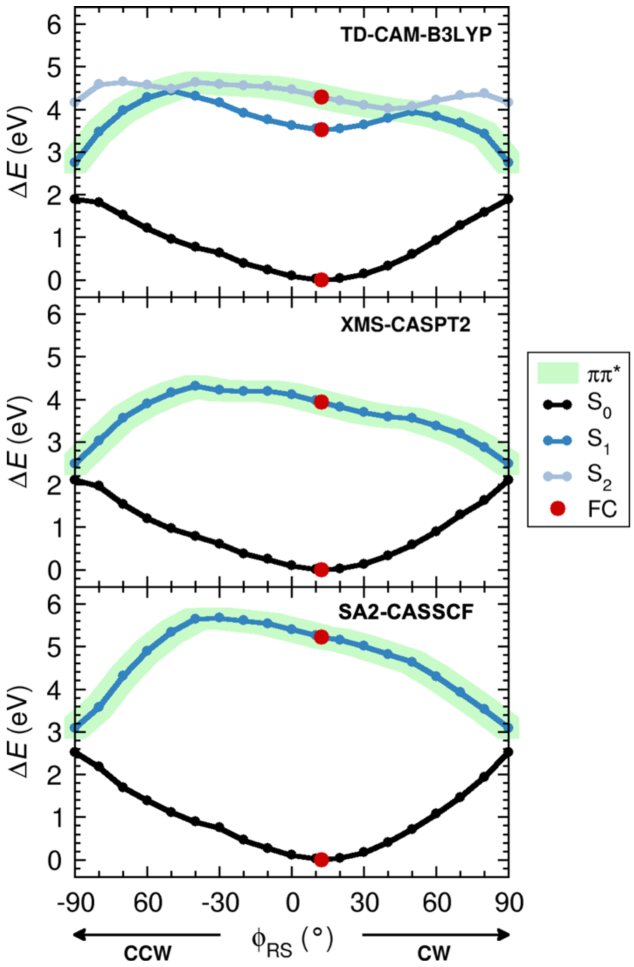

Fig. S3 S_0, S_1 and S_2 energy profiles for changing the ϕ_{RS} dihedral angle of **PG-3** calculated at different levels of theory, with the ππ* state highlighted in light-green color. A full red circle indicates the value of ϕ_{RS} at the vertically excited FC point (i.e., at the S_0 minimum).

4. Size of the active space

In order to validate the choice to use a small (4,4) active space for the multiconfigurational calculations, some of these calculations were repeated with a larger (6,6) active space containing also two π-electrons and two π/π*-orbitals associated with the barrelene propeller.

First, this was done for the XMS-CASPT2 calculations in Fig. S3, producing results that in Fig. S4 are compared to the original results. Here, it may again be noted that, in the FC region, the S_1 state calculated with the (6,6) active space is a dark CT state formed upon excitation from a π orbital of the propeller to a π* orbital of the rotor-stator Schiff-base core. As for the bright ππ* state, it is encouraging that the two active spaces yield almost indistinguishable energy profiles for both directions of rotor rotation. For example, the difference in the projected energy barriers for the disfavored CCW rotor rotation is only 0.01 eV. Thus, the initial reaction dynamics for the bright ππ* state predicted with the (4,4) active space (for which this state is always S_1) is similar to that predicted with the (6,6) active space (for which this state is S_2 in the FC region).

Second, also the CASSCF geometry optimization of the S_0 minimum of **PG-3** shown in Fig. 3 of the main text was repeated with the (6,6) active space. Here, it is of special interest to assess how much the ϕ_{RS} and ϕ_{PS} dihedral

angles vary with the choice of active space, which is done in Fig. S5. Accordingly, this variation is found to be very small (of the order of 1° only). Overall, then, the results in Fig. S4 and S5 support the use of a small (4,4) active space for the multiconfigurational calculations.

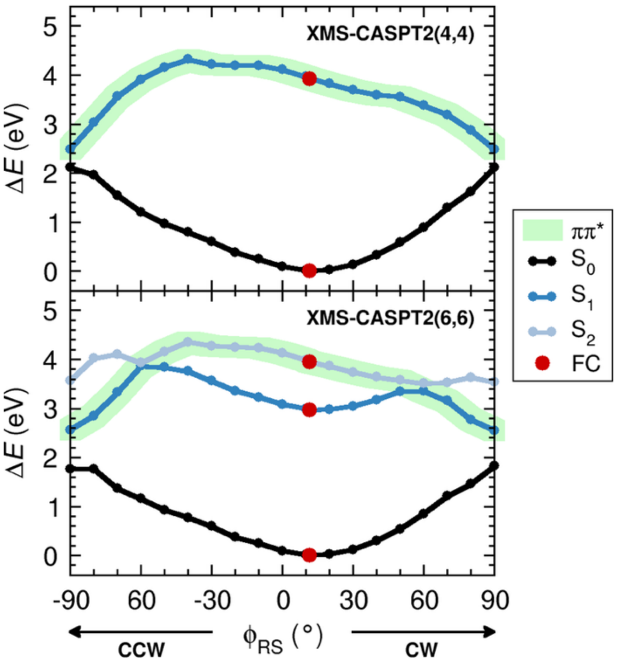

Fig. S4 S_0, S_1 and S_2 energy profiles for changing the ϕ_{RS} dihedral angle of **PG-3** calculated at the XMS-CASPT2 level of theory with (4,4) and (6,6) active spaces. The ππ* state is highlighted in light-green color. A full red circle indicates the value of ϕ_{RS} at the vertically excited FC point (i.e., at the S_0 minimum).

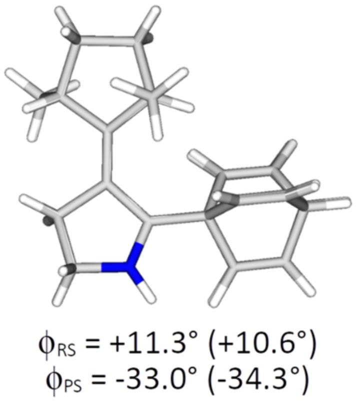

Fig. S5 Comparison of ϕ_{RS} and ϕ_{PS} dihedral angles at the S_0 minimum of **PG-3** calculated at the CASSCF level of theory with (4,4) and (6,6) active spaces. The values obtained with the (6,6) active space are given in parentheses.

5. MD simulations

As a complement to Fig. 4 of the main text, which plots all possible $\Delta\phi_{RS}$ and $\Delta\phi_{PS}$ values among the 20 NAMD trajectories at each time step, Fig. S6 instead plots the $\Delta\phi_{RS}$ and $\Delta\phi_{PS}$ values for the individual trajectories separately. This reaffirms the conclusion that forward photogearing is much more favored than reverse photogearing, with all green-colored trajectories starting to approach the top-right plotting area representing

forward photogearing, and no single trajectory moving toward the bottom-left area representing reverse photogearing. Furthermore, while the forward photogearing observed in the green-colored trajectories is asynchronous, with the propeller rotation (i.e., the motion along the $\Delta\phi_{PS}$ coordinate) mostly taking place after decay to the S_0 state at quite large $\Delta\phi_{RS}$ values, some propeller rotation is certainly possible already in the S_1 state. Indeed, this can be seen from two of the yellow-colored trajectories in Fig. S6. Notably, the corresponding trajectories, which are encircled, show propeller rotation in the same forward direction as the green-colored trajectories.

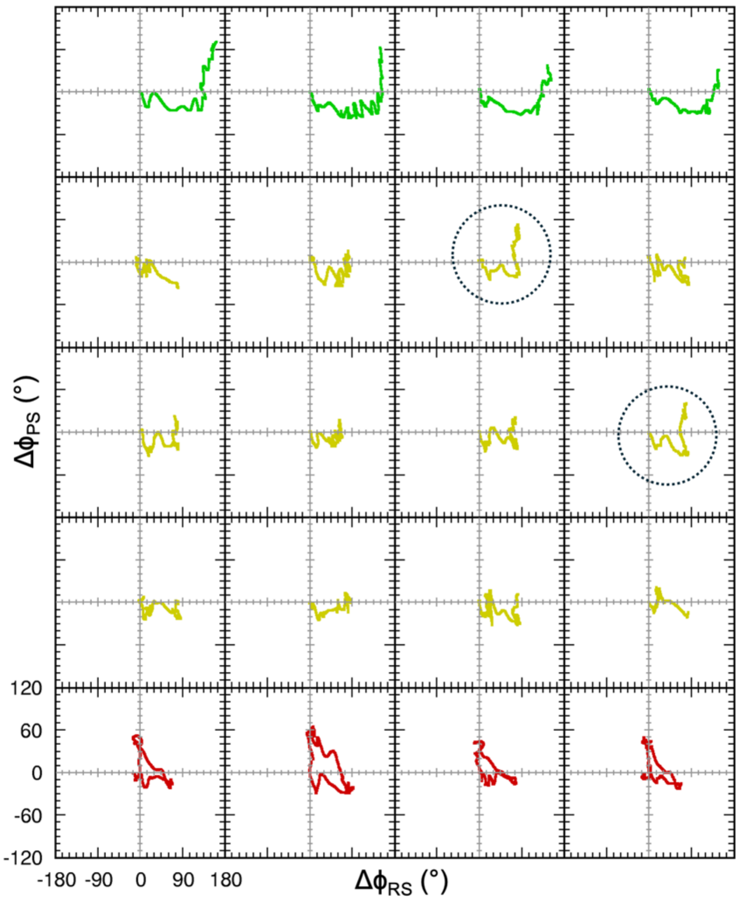

Fig. S6 Key structural changes along each individual NAMD trajectory calculated for **PG-3**. The color-coding used for plotting the trajectories is explained in the main text. Each of the 20 plots shows the changes in the ϕ_{RS} and ϕ_{PS} dihedral angles ($\Delta\phi_{RS}$ and $\Delta\phi_{PS}$) relative to the unique ϕ_{RS} and ϕ_{PS} values at the starting point of the trajectory in question. Accordingly, each trajectory begins at $\Delta\phi_{RS} = 0°$ and $\Delta\phi_{PS} = 0°$. Notice that the values along the abscissa and ordinate axes are consistently plotted using the scale employed for the bottom-left graph. The two encircled, yellow-colored trajectories are trajectories in which some propeller rotation occurs already in the S_1 state.

As already indicated in the main text, CASSCF-based NAMD simulations are expensive, which is why they were limited to 500 fs. While this time frame is sufficient for forward photogearing and decay to the S_0 state to be observed in the four green-colored trajectories in Fig. 4 of the main text, these trajectories were then propagated adiabatically in the S_0 state for an additional 500 fs. Pleasingly, these results, which are given in Fig. S7, show that the subsequent S_0 dynamics continues the forward photogearing to complete, in each case, a full CCW propeller rotation ($\Delta\phi_{PS} = +120°$). In order to clearly highlight this finding, an animation of one of the four total trajectories in Fig. S7 is provided as a supporting multimedia file, as described in Section 7 of this document.

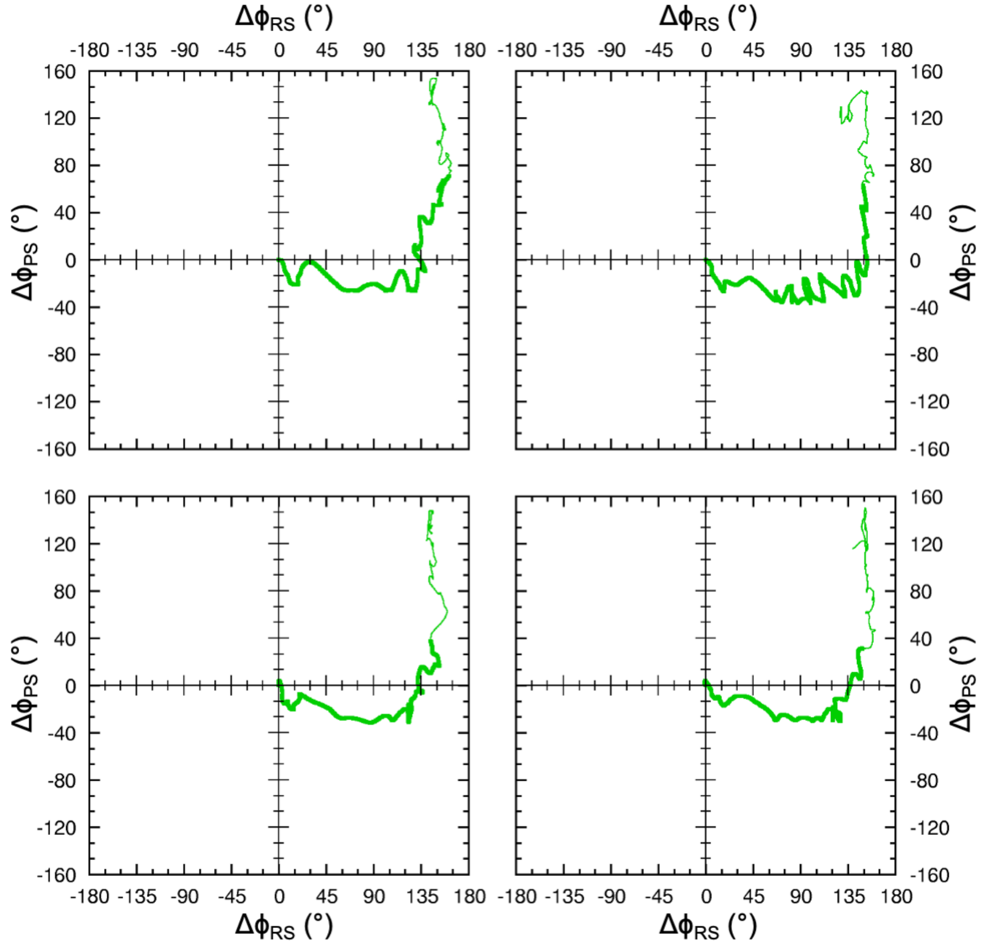

Fig. S7 Continuation of the four forward photogearing NAMD trajectories in Fig. 4 of the main text by adiabatic MD simulations in the S_0 state for an additional 500 fs. The initial NAMD portions of the trajectories are plotted in bold green font, and the subsequent adiabatic MD portions are plotted in normal green font.

Besides these simulations, also the four red-colored trajectories in Fig. 4 of the main text representing FC repopulation (i.e., decay to the S_0 state and reformation of the parent S_0 minimum) were propagated adiabatically in the S_0 state for an additional 500 fs. The corresponding trajectories are presented in Fig. S8. Here, it is particularly encouraging that propeller slippage (i.e., pronounced motion along the $\Delta\phi_{PS}$ coordinate) continues to be absent during the extended simulations. Of course, as further discussed in Section 6 of this document, this does not necessarily mean that propeller slippage is an irrelevant process!

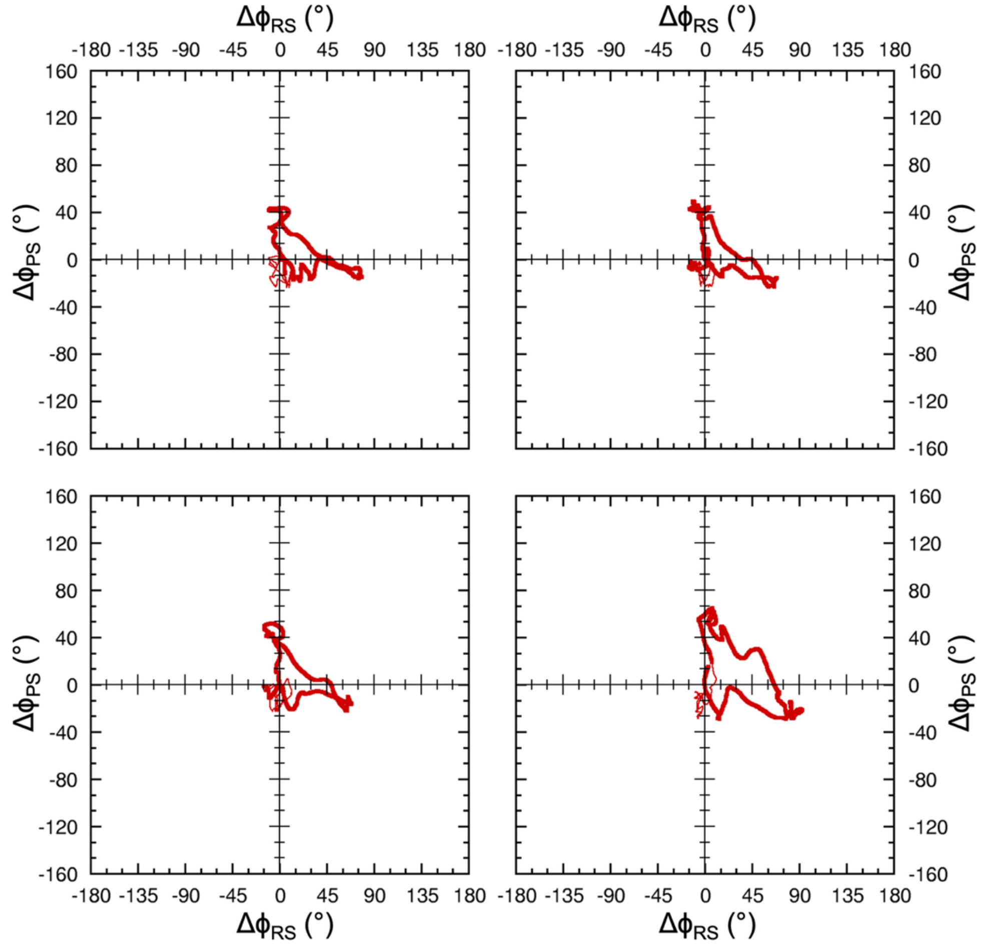

Fig. S8 Continuation of the four FC repopulation NAMD trajectories in Fig. 4 of the main text by adiabatic MD simulations in the S_0 state for an additional 500 fs. The initial NAMD portions of the trajectories are plotted in bold red font, and the subsequent adiabatic MD portions are plotted in normal red font.

6. Thermal barriers for rotor and propeller slippage processes

As discussed in the main text, and shown in Table S2, CAM-B3LYP and M06-2X calculations predict **PG-3** to exhibit free-energy barriers that are large (33–40 kcal mol^{-1}) for rotor slippage and small (7–8 kcal mol^{-1}) for propeller slippage. Thus, from the viewpoint of photogearing efficiency, it is important to identify strategies to increase the latter values while keeping the key structural features of the **PG-3** template intact. To this end, the propeller-slippage barrier was also calculated for the **PG-3** derivatives **PG-3-Me** and **PG-3-Ph** depicted in Fig. S9. Here, **PG-3-Me** and **PG-3-Ph** represent strategies to increase the steric repulsion between either the propeller and the stator (**PG-3-Me**), or the propeller and the rotor (**PG-3-Ph**). In **PG-3-Me**, this is achieved by replacing the protonated Schiff base of the stator with a methylated ditto, whereas in **PG-3-Ph**, the idea is to increase the size of the propeller blades by making them contain phenyl groups. Thereby, the propeller takes the form of the full triptycene molecule often employed by thermally driven molecular gears.[13–15] From the results of these calculations, which are also included in Table S2, it can be seen that both strategies are successful, but particularly the latter one, raising the propeller-slippage barrier to an appreciable 20–23 kcal mol^{-1} for **PG-3-Ph**.

Table S2 Free-energy barriers of rotor and propeller slippage processes for **PG-3** and derivatives thereof calculated with the CAM-B3LYP and M06-2X density functionals [a]

	**PG-3** Rotor slippage	**PG-3** Propeller slippage	**PG-3-Me** Propeller slippage	**PG-3-Ph** Propeller slippage
CAM-B3LYP [b]	33.4 (33.8)	7.3 (6.7)	10.2 (9.7)	19.8 (18.7)
M06-2X [b]	39.5 (39.8)	8.2 (8.0)	11.3 (10.5)	22.5 (21.5)

[a] Barriers are given in kcal mol^{-1}. The **PG-3** derivatives **PG-3-Me** and **PG-3-Ph** are shown in Fig. S9. [b] Values in parentheses are barriers given in terms of electronic energies.

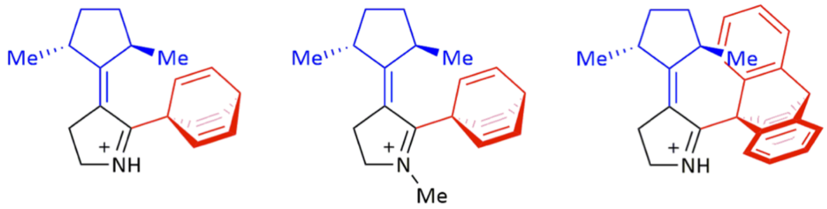

PG-3 **PG-3-Me** **PG-3-Ph**

Fig. S9 Chemical structures of **PG-3-Me** and **PG-3-Ph** compared to **PG-3**.

7. Description of supplementary multimedia file

The supplementary multimedia file **PHOTOGEAR-TRAJ.mp4** provides an animation of one of the full forward photogearing trajectories in Fig. S7. For ease of interpretation, all hydrogen atoms are hidden in the animation. Furthermore, two carbon atoms are highlighted in red color – one belonging to the rotor and the other belonging to the propeller.

8. References

1 T. Yanai, D. P. Tew and N. C. Handy, *Chem. Phys. Lett.*, 2004, **393**, 51–57.
2 T. H. Dunning, Jr., *J. Chem. Phys.*, 1989, **90**, 1007–1023.
3 M. E. Casida and M. Huix-Rotllant, *Annu. Rev. Phys. Chem.*, 2012, **63**, 287–323.
4 B. O. Roos, P. R. Taylor and P. E. M. Siegbahn, *Chem. Phys.*, 1980, **48**, 157–173.
5 K. Andersson, P.-Å. Malmqvist and B. O. Roos, *J. Chem. Phys.*, 1992, **96**, 1218–1226.
6 T. Shiozaki, W. Győrffy, P. Celani and H.-J. Werner, *J. Chem. Phys.*, 2011, **135**, 081106.
7 M. J. Frisch, G. W. Trucks, H. B. Schlegel, G. E. Scuseria, M. A. Robb, J. R. Cheeseman, G. Scalmani, V. Barone, G. A. Petersson, H. Nakatsuji, X. Li, M. Caricato, A. V. Marenich, J. Bloino, B. G. Janesko, R. Gomperts, B. Mennucci, H. P. Hratchian, J. V. Ortiz, A. F. Izmaylov, J. L. Sonnenberg, D. Williams-Young, F. Ding, F. Lipparini, F. Egidi, J. Goings, B. Peng, A. Petrone, T. Henderson, D. Ranasinghe, V. G. Zakrzewski, J. Gao, N. Rega, G. Zheng, W. Liang, M. Hada, M. Ehara, K. Toyota, R. Fukuda, J. Hasegawa, M. Ishida, T. Nakajima, Y. Honda, O. Kitao, H. Nakai, T. Vreven, K. Throssell, J. A. Montgomery, Jr., J. E. Peralta, F. Ogliaro, M. J. Bearpark, J. J. Heyd, E. N. Brothers, K. N. Kudin, V. N. Staroverov, T. A. Keith, R. Kobayashi, J. Normand, K. Raghavachari, A. P. Rendell, J. C. Burant, S. S. Iyengar, J. Tomasi, M. Cossi, J. M. Millam, M. Klene, C. Adamo, R. Cammi, J. W. Ochterski, R. L. Martin, K. Morokuma, O. Farkas, J. B. Foresman and D. J. Fox, *Gaussian 16*, *Revision C.01*, Gaussian, Inc., Wallingford CT, 2016.
8 I. F. Galván, M. Vacher, A. Alavi, C. Angeli, F. Aquilante, J. Autschbach, J. J. Bao, S. I. Bokarev, N. A. Bogdanov, R. K. Carlson, L. F. Chibotaru, J. Creutzberg, N. Dattani, M. G. Delcey, S. S. Dong, A. Dreuw, L. Freitag, L. M. Frutos, L. Gagliardi, F. Gendron, A. Giussani, L. González, G. Grell, M. Guo, C. E. Hoyer, M. Johansson, S. Keller, S. Knecht, G. Kovačević, E. Källman, G. L. Manni, M. Lundberg, Y. Ma, S. Mai, J. P. Malhado, P. Å. Malmqvist, P. Marquetand, S. A. Mewes, J. Norell, M. Olivucci, M. Oppel, Q. M. Phung, K. Pierloot, F. Plasser, M. Reiher, A. M. Sand, I. Schapiro, P. Sharma, C. J. Stein, L. K. Sørensen, D. G. Truhlar, M. Ugandi, L. Ungur, A. Valentini, S. Vancoillie, V. Veryazov, O. Weser, T. A. Wesołowski, P.-O. Widmark, S. Wouters, A. Zech, J. P. Zobel and R. Lindh, *J. Chem. Theory Comput.*, 2019, **15**, 5925–5964.
9 J. C. Tully, *J. Chem. Phys.*, 1990, **93**, 1061–1071.
10 F. Aquilante, J. Autschbach, R. K. Carlson, L. F. Chibotaru, M. G. Delcey, L. De Vico, I. F. Galván, N. Ferré, L. M. Frutos, L. Gagliardi, M. Garavelli, A. Giussani, C. E. Hoyer, G. Li Manni, H. Lischka, D. Ma, P. Å. Malmqvist, T. Müller, A. Nenov, M. Olivucci, T. B. Pedersen, D. Peng, F. Plasser, B. Pritchard, M. Reiher, I. Rivalta, I. Schapiro, J. Segarra-Martí, M. Stenrup, D. G. Truhlar, L. Ungur, A. Valentini, S. Vancoillie, V. Veryazov, V. P. Vysotskiy, O. Weingart, F. Zapata and R. Lindh, *J. Comput. Chem.*, 2016, **37**, 506–541.
11 Y. Zhao and D. G. Truhlar, *Theor. Chem. Acc.*, 2008, **120**, 215–241.
12 H. P. Hratchian and H. B. Schlegel, *J. Chem. Phys.*, 2004, **120**, 9918–9924.
13 D. K. Frantz, A. Linden, K. K. Baldridge and J. S. Siegel, *J. Am. Chem. Soc.*, 2012, **134**, 1528–1535.
14 X. Jiang, S. Yang, M. J. Jellen, K. N. Houk and M. Garcia-Garibay, *Org. Lett.*, 2020, **22**, 4049–4052.
15 M. J. Jellen, I. Liepuoniute, M. Jin, C. G. Jones, S. Yang, X. Jiang, H. M. Nelson, K. N. Houk and M. Garcia-Garibay, *J. Am. Chem. Soc.*, 2021, **143**, 7740–7747.

9. Cartesian coordinates and energies of optimized geometries

Cartesian coordinates of geometries optimized at the indicated level of theory are given in Å.

Electronic energies (E) of geometries optimized at the indicated level of theory are given in a.u.

The single imaginary vibrational frequency (ω) of a transition structure (TS) for rotor or propeller slippage calculated at the indicated level of theory is given in cm^{-1}.

PG-3 (44 atoms)
S_0 minimum
CAM-B3LYP/cc-pVDZ
$E(S_0) = -792.713946020$

C	0.413869	3.343835	0.657062
N	−0.768887	2.490963	0.486697
C	−0.503574	1.247332	0.176636
C	0.936322	1.091373	0.037331
C	1.493216	2.511373	−0.039792
H	0.605977	3.480941	1.732539
H	2.470205	2.611888	0.445981
C	1.693100	−0.036626	0.066544
C	3.176574	−0.028782	−0.274346
H	3.707720	0.701969	0.356867
C	1.292016	−1.432460	0.486656
H	0.284532	−1.706387	0.153406
C	2.382126	−2.316115	−0.138982
H	2.456299	−3.295070	0.353236
H	2.155175	−2.500216	−1.200711
C	3.651805	−1.469987	−0.001274
H	4.445500	−1.774405	−0.696118
H	4.065366	−1.557514	1.013223
C	1.335792	−1.520451	2.026242
H	0.649035	−0.805872	2.498299
H	1.048156	−2.533667	2.341321
H	2.342059	−1.318579	2.419910
C	3.395320	0.358239	−1.746927
H	3.012579	1.359726	−1.984129
H	4.470828	0.349382	−1.972753
H	2.905008	−0.359957	−2.421415
H	−1.717861	2.821340	0.633772
H	1.600666	2.838719	−1.085281
H	0.235973	4.325629	0.202950
C	−1.633925	0.287920	−0.064008
C	−1.385376	−0.715532	−1.212557
H	−0.486071	−0.661556	−1.822710
C	−2.387392	−1.568896	−1.377857
H	−2.423101	−2.347324	−2.138631
C	−1.956229	−0.560573	1.191738
H	−1.416531	−0.418971	2.125133
C	−2.952924	−1.412829	0.994731
H	−3.343443	−2.093528	1.750175
C	−2.973944	0.973593	−0.435849
H	−3.060931	2.044882	−0.614393
C	−3.966436	0.111278	−0.609553
H	−4.979707	0.381633	−0.902992
C	−3.554994	−1.350162	−0.408682
H	−4.377301	−2.054996	−0.557242

PG-3 (44 atoms)
TS for rotor slippage
CAM-B3LYP/cc-pVDZ
$E(S_0)$ = −792.655150517
ω = 226.2389i

C	−0.252343	3.375195	0.084548
N	0.882792	2.504952	0.385961
C	0.506005	1.190762	0.194333
C	−0.841843	1.077549	0.120204
C	−1.467004	2.467428	0.335288
H	−0.227723	3.698002	−0.971320
H	−2.302759	2.696313	−0.340406
C	−1.728817	−0.055435	0.034487
C	−2.403262	−0.692436	1.176189
H	−3.209287	0.099162	1.221388
C	−2.216882	−0.645521	−1.223877
H	−1.325476	−1.318331	−1.366196
C	−3.400328	−1.545684	−0.835088
H	−4.329185	−0.953588	−0.884242
H	−3.516679	−2.398614	−1.514469
C	−3.088290	−1.944136	0.607341
H	−2.376279	−2.785393	0.641238
H	−3.971828	−2.235586	1.187990
C	−2.340682	0.238065	−2.455635
H	−1.432556	0.833762	−2.615377
H	−2.510630	−0.381478	−3.345821
H	−3.197483	0.920359	−2.352547
C	−1.726325	−0.757770	2.533464
H	−1.241563	0.192908	2.792826
H	−2.463945	−0.997318	3.310420
H	−0.960441	−1.546725	2.543517
H	1.798184	2.779396	0.043100
H	−1.834606	2.560031	1.372234
H	−0.260865	4.267508	0.723302
C	1.579927	0.155220	0.052023
C	1.150348	−1.304305	0.255131
H	0.148374	−1.575127	0.584484
C	2.135284	−2.177027	0.075851
H	2.048708	−3.256020	0.196957
C	2.209225	0.203754	−1.362795
H	1.842649	0.904683	−2.112730
C	3.188720	−0.674031	−1.531269
H	3.759500	−0.810351	−2.448914
C	2.764497	0.359468	1.026144
H	2.740473	1.150078	1.774252
C	3.744149	−0.515448	0.843092
H	4.666329	−0.554873	1.421099
C	3.470545	−1.519423	−0.285264
H	4.279982	−2.240815	−0.427507

PG-3 (44 atoms)
TS for propeller slippage
CAM-B3LYP/cc-pVDZ
$E(S_0) = -792.703234265$
$\omega = 37.6087i$

C	−0.208771	3.174761	−0.705091
N	0.865606	2.197321	−0.561123
C	0.502960	0.974610	−0.249444
C	−0.953715	0.956110	−0.087052
C	−1.383626	2.427595	−0.082674
H	−0.352945	3.398196	−1.773305
H	−2.309101	2.596138	−0.642789
C	−1.871657	−0.046794	−0.011484
C	−3.345669	0.263751	0.202264
H	−3.654620	1.136533	−0.389803
C	−1.712828	−1.547101	−0.177876
H	−0.987863	−1.932943	0.547175
C	−3.108057	−2.127909	0.128151
H	−3.287819	−3.062836	−0.418933
H	−3.195331	−2.359948	1.199637
C	−4.072835	−1.008230	−0.254543
H	−5.062930	−1.109457	0.209483
H	−4.224269	−0.985854	−1.344506
C	−1.264693	−1.891992	−1.607772
H	−0.312300	−1.413408	−1.876025
H	−1.140326	−2.979556	−1.710349
H	−2.017835	−1.569197	−2.341626
C	−3.612286	0.574166	1.686978
H	−3.048268	1.449533	2.037440
H	−4.681805	0.782876	1.831088
H	−3.344805	−0.275977	2.331244
H	1.845910	2.432005	−0.701634
H	−1.555520	2.774018	0.947565
H	0.059308	4.104654	−0.188987
C	1.692553	0.061890	−0.013362
C	1.587611	−1.458609	0.187910
H	0.661715	−2.004645	0.099882
C	2.758636	−2.030045	0.440931
H	2.898948	−3.097504	0.603040
C	2.743964	0.232363	−1.146241
H	2.491628	0.730436	−2.082296
C	3.905949	−0.347413	−0.878140
H	4.760782	−0.365722	−1.552953
C	2.397661	0.582016	1.279262
H	1.930202	1.341119	1.904682
C	3.563091	−0.003294	1.512284
H	4.207787	0.217110	2.362181
C	3.941901	−1.059492	0.475123
H	4.899080	−1.545122	0.682299

PG-3 (44 atoms)

S_0 minimum

M06-2X/cc-pVDZ

$E(S_0) = -792.836707480$

C	0.438490	3.355850	0.646046
N	−0.757190	2.513928	0.483876
C	−0.501206	1.269128	0.173749
C	0.937061	1.104849	0.022426
C	1.500199	2.519193	−0.077631
H	0.646593	3.473031	1.719895
H	2.489914	2.616312	0.381828
C	1.672894	−0.036106	0.069883
C	3.153748	−0.059390	−0.275532
H	3.706409	0.662984	0.346259
C	1.247525	−1.415654	0.513262
H	0.224526	−1.676246	0.219006
C	2.304399	−2.327329	−0.129356
H	2.361065	−3.306674	0.363290
H	2.054957	−2.500745	−1.187567
C	3.598578	−1.512333	−0.010113
H	4.369846	−1.833164	−0.721966
H	4.026329	−1.612946	0.996425
C	1.344792	−1.468080	2.052669
H	0.696024	−0.719131	2.525737
H	1.042822	−2.465392	2.401646
H	2.370645	−1.280484	2.399707
C	3.348246	0.315898	−1.754860
H	2.974663	1.322675	−1.983986
H	4.417331	0.284780	−2.004322
H	2.825127	−0.399493	−2.407288
H	−1.703183	2.849500	0.646810
H	1.572323	2.841664	−1.127315
H	0.261562	4.342192	0.204633
C	−1.621197	0.301270	−0.061391
C	−1.359015	−0.685879	−1.222553
H	−0.453160	−0.614768	−1.823215
C	−2.347762	−1.556794	−1.388540
H	−2.373558	−2.333801	−2.150364
C	−1.913759	−0.564393	1.190679
H	−1.369319	−0.416622	2.120750
C	−2.897490	−1.434085	0.991312
H	−3.270451	−2.129672	1.741304
C	−2.973063	0.969660	−0.414330
H	−3.076913	2.041113	−0.582760
C	−3.952498	0.090178	−0.590212
H	−4.972071	0.343746	−0.874759
C	−3.512983	−1.366464	−0.407862
H	−4.322909	−2.083468	−0.556754

PG-3 (44 atoms)
TS for rotor slippage
M06-2X/cc-pVDZ
$E(S_0)$ = −792.769955410
ω = 294.6752i

C	−0.305059	3.415129	0.251417
N	0.850694	2.542994	0.449207
C	0.496489	1.253405	0.115678
C	−0.853029	1.113120	0.036292
C	−1.495069	2.461517	0.434657
H	−0.305936	3.831535	−0.770053
H	−2.361345	2.761450	−0.165875
C	−1.693085	−0.056702	−0.013207
C	−2.065839	−0.858916	1.178029
H	−2.401853	−0.113564	1.925885
C	−2.457451	−0.502840	−1.197419
H	−1.844190	−0.431169	−2.107054
C	−2.937039	−1.913836	−0.818200
H	−3.828728	−2.214416	−1.382048
H	−2.143073	−2.644027	−1.037907
C	−3.173715	−1.816382	0.695805
H	−3.116341	−2.789297	1.198641
H	−4.165929	−1.394673	0.908826
C	−3.640126	0.503592	−1.362069
H	−3.277072	1.477049	−1.710082
H	−4.313510	0.084222	−2.120878
H	−4.206704	0.635612	−0.429734
C	−0.856290	−1.577667	1.830843
H	−0.019241	−0.889531	2.004128
H	−1.191496	−1.982314	2.793650
H	−0.511535	−2.402950	1.195447
H	1.757458	2.871849	0.129965
H	−1.808493	2.411435	1.491704
H	−0.322503	4.238782	0.974726
C	1.585597	0.247554	−0.088454
C	1.140547	−1.117844	−0.623898
H	0.110450	−1.315293	−0.926912
C	2.137665	−1.988105	−0.753809
H	2.042254	−3.004698	−1.131374
C	2.672683	0.721040	−1.086998
H	2.572997	1.660305	−1.630688
C	3.667892	−0.148128	−1.220013
H	4.527196	−0.023777	−1.876494
C	2.348212	−0.032956	1.228194
H	2.071520	0.485884	2.145588
C	3.336916	−0.907177	1.081097
H	4.000647	−1.245889	1.874806
C	3.500865	−1.408986	−0.358461
H	4.319588	−2.122381	−0.475618

PG-3 (44 atoms)
TS for propeller slippage
M06-2X/cc-pVDZ
$E(S_0)$ = −792.823979136
ω = 37.3972i

C	−0.203180	3.192550	−0.665576
N	0.864296	2.205041	−0.523734
C	0.493362	0.978682	−0.236859
C	−0.965343	0.966072	−0.088882
C	−1.400768	2.434678	−0.095187
H	−0.316120	3.445668	−1.729576
H	−2.300406	2.599046	−0.696752
C	−1.876271	−0.042621	−0.011634
C	−3.354459	0.254733	0.168186
H	−3.664341	1.125483	−0.425425
C	−1.691086	−1.541203	−0.138857
H	−0.981446	−1.898198	0.615754
C	−3.088692	−2.134977	0.133221
H	−3.238749	−3.077335	−0.409279
H	−3.207662	−2.352031	1.203995
C	−4.053529	−1.030089	−0.297858
H	−5.056126	−1.135583	0.136245
H	−4.165042	−1.022193	−1.392332
C	−1.206805	−1.890845	−1.556978
H	−0.278489	−1.363577	−1.821996
H	−1.028117	−2.972320	−1.638778
H	−1.971425	−1.615183	−2.298038
C	−3.636919	0.550772	1.653700
H	−3.084272	1.430701	2.009988
H	−4.709997	0.741985	1.789924
H	−3.357625	−0.302426	2.288274
H	1.849888	2.435061	−0.647737
H	−1.616879	2.773275	0.928608
H	0.057761	4.102501	−0.113681
C	1.678598	0.056334	−0.013840
C	1.585762	−1.469746	0.162615
H	0.671517	−2.031341	0.050575
C	2.764914	−2.031842	0.409686
H	2.916994	−3.100178	0.550463
C	2.728659	0.252555	−1.144664
H	2.467119	0.763344	−2.071429
C	3.898192	−0.318677	−0.883300
H	4.754174	−0.319260	−1.556089
C	2.376191	0.563899	1.288168
H	1.895126	1.307118	1.922359
C	3.549004	−0.013181	1.514036
H	4.190301	0.199312	2.367898
C	3.939644	−1.050416	0.460907
H	4.900101	−1.529216	0.661862

PG-3 (44 atoms)

S_0 minimum

CASSCF/cc-pVDZ

$E(S_0) = -787.89650548$

$E(S_1) = -787.70263886$

C	0.41107555	3.33510414	0.64858918
N	−0.77006964	2.47431410	0.48786497
C	−0.51202468	1.24070178	0.17685784
C	0.93057179	1.08476784	0.02635650
C	1.48063318	2.51004307	−0.06579882
H	0.60341682	3.46533270	1.71255470
H	2.45311452	2.61875701	0.40385749
C	1.70313072	−0.03889602	0.06446802
C	3.20080333	−0.01990062	−0.27623124
H	3.72726596	0.68717988	0.36748506
C	1.31826431	−1.44755876	0.50599853
H	0.32821107	−1.73905794	0.17214477
C	2.41147899	−2.31724533	−0.13196384
H	2.50127406	−3.29011871	0.35089704
H	2.17725646	−2.49957261	−1.18379322
C	3.67725049	−1.46394815	−0.00782983
H	4.45824460	−1.76609362	−0.70457592
H	4.09918134	−1.55077700	0.99349233
C	1.36472984	−1.54012054	2.04217334
H	0.67250962	−0.84376823	2.51411281
H	1.09419707	−2.54911266	2.35654773
H	2.35847425	−1.32439567	2.43603903
C	3.44158625	0.37576568	−1.74109925
H	3.07696729	1.37538961	−1.97447835
H	4.51122449	0.35922850	−1.95311385
H	2.95642431	−0.32493018	−2.42416348
H	−1.70378760	2.80476485	0.63932035
H	1.56377825	2.83339420	−1.10294937
H	0.21653743	4.30449969	0.20007231
C	−1.66184287	0.27844854	−0.06074413
C	−1.42325646	−0.72296834	−1.21704804
H	−0.53090885	−0.68659159	−1.82187823
C	−2.42544013	−1.56108073	−1.38130484
H	−2.46597459	−2.33233928	−2.13610387
C	−1.99536401	−0.56440974	1.19493237
H	−1.46499196	−0.43264635	2.12437791
C	−2.99349628	−1.40026916	0.99937289
H	−3.39008144	−2.06912269	1.74897930
C	−2.99678031	0.98299790	−0.43983705
H	−3.07948529	2.04531590	−0.61694822
C	−3.99044628	0.13640166	−0.61195495
H	−4.99173206	0.41638129	−0.90393619
C	−3.59121617	−1.33077377	−0.40894413
H	−4.41411239	−2.02153066	−0.55755064

PG-3 (44 atoms)

S_1/S_0 CI for rotor rotation

CASSCF/cc-pVDZ

$E(S_0) = -787.80354853$

$E(S_1) = -787.80136500$

C	0.12589444	3.04126543	1.57086854
N	−0.89313602	2.38697659	0.76057088
C	−0.53739524	1.15016607	0.38223341
C	0.78640344	0.91115785	0.68330242
C	1.37623441	2.18953854	1.30296916
H	−0.16248290	3.01387915	2.62306823
H	1.94962251	2.01716571	2.21139091
C	1.67701332	−0.16940175	0.34373934
C	2.50765711	−0.21235865	−0.91215258
H	2.99091252	0.76913703	−0.98193592
C	2.05215616	−1.29562414	1.26706194
H	1.13752209	−1.73165587	1.67470492
C	2.83385404	−2.26114287	0.35704313
H	3.53199157	−2.88153577	0.91706616
H	2.14051953	−2.93589331	−0.15015826
C	3.52448502	−1.34570211	−0.65989019
H	3.79271343	−1.86394418	−1.57919303
H	4.44631328	−0.93798032	−0.24210070
C	2.88000544	−0.78484866	2.47108259
H	2.29053623	−0.12680459	3.10707504
H	3.17992031	−1.64708051	3.06599393
H	3.78339831	−0.25988340	2.15745490
C	1.68573480	−0.38934434	−2.20584276
H	0.91481401	0.37284489	−2.30360911
H	2.36441344	−0.29773854	−3.05340399
H	1.21406264	−1.36991937	−2.25189549
H	−1.85620094	2.65002117	0.82172499
H	2.04083903	2.68060009	0.58648800
H	0.25088499	4.07986834	1.27369356
C	−1.59884090	0.29552912	−0.27014925
C	−1.25613523	−1.18441718	−0.52376189
H	−0.32544849	−1.63022365	−0.21337559
C	−2.22967206	−1.84851230	−1.11361675
H	−2.19682321	−2.89753921	−1.36892798
C	−2.90989717	0.25312223	0.56354783
H	−2.97570215	0.70351492	1.54374411
C	−3.87724436	−0.41366256	−0.03090578
H	−4.86244314	−0.57478431	0.38158575
C	−2.01056230	0.87475354	−1.64876878
H	−1.52693317	1.75272480	−2.05196886
C	−2.98073157	0.19719214	−2.22579448
H	−3.41409917	0.43182741	−3.18705670
C	−3.47314062	−0.99377654	−1.39423816
H	−4.27184041	−1.54881185	−1.87506846

PG-3-Me (47 atoms)
S_0 minimum
CAM-B3LYP/cc-pVDZ
$E(S_0)$ = –832.009480851

C	0.262812	3.194780	0.296583
N	–0.867682	2.245302	0.343593
C	–0.514480	1.026696	0.010127
C	0.927243	0.998857	–0.242274
C	1.302266	2.443982	–0.530330
H	0.597610	3.390918	1.328129
H	2.326462	2.692335	–0.233414
C	1.808731	–0.001286	–0.003031
C	3.288317	0.119999	–0.345464
H	3.715169	1.021568	0.122676
C	1.571121	–1.321675	0.701872
H	0.619507	–1.789575	0.426332
C	2.779188	–2.170134	0.277682
H	2.964952	–3.005321	0.966417
H	2.601292	–2.602750	–0.719494
C	3.930151	–1.161099	0.223663
H	4.771216	–1.504453	–0.393206
H	4.327720	–0.978661	1.232320
C	1.559655	–1.082980	2.223880
H	0.737095	–0.418096	2.522982
H	1.429840	–2.041768	2.746199
H	2.497228	–0.630657	2.578378
C	3.485829	0.230139	–1.865273
H	2.979137	1.108423	–2.289261
H	4.557059	0.314611	–2.096817
H	3.099737	–0.662328	–2.381070
H	1.185716	2.687089	–1.598075
H	–0.081344	4.138382	–0.145384
C	–1.527802	–0.072028	–0.168798
C	–1.071231	–1.253478	–1.056918
H	–0.116979	–1.230899	–1.576657
C	–1.986434	–2.207724	–1.161159
H	–1.875657	–3.114410	–1.753890
C	–1.972050	–0.686348	1.178372
H	–1.548385	–0.348322	2.122816
C	–2.886618	–1.637546	1.038225
H	–3.329052	–2.197078	1.861668
C	–2.833205	0.405386	–0.867533
H	–2.927297	1.405781	–1.283829
C	–3.745110	–0.551300	–0.970842
H	–4.717728	–0.433663	–1.446600
C	–3.288405	–1.902308	–0.410711
H	–4.041310	–2.688537	–0.511484
C	–2.144320	2.725509	0.858593
H	–2.617978	3.400082	0.131433
H	–2.815550	1.893406	1.086752
H	–1.938628	3.292158	1.777944

PG-3-Me (47 atoms)
TS for propeller slippage
CAM-B3LYP/cc-pVDZ
$E(S_0) = -831.994697345$
$\omega = 37.9128i$

C	−0.129173	3.139392	−0.392670
N	0.925511	2.131530	−0.255356
C	0.500731	0.882961	−0.181392
C	−0.974642	0.878670	−0.208925
C	−1.408778	2.330843	−0.422700
H	0.056539	3.722447	−1.306026
H	−1.920269	2.429023	−1.390587
C	−1.946558	−0.069468	−0.064633
C	−3.400780	0.371744	0.104432
H	−3.652807	1.189747	−0.584239
C	−1.925757	−1.587620	−0.100659
H	−1.183632	−1.997212	0.591902
C	−3.341780	−2.015770	0.325631
H	−3.607261	−2.999857	−0.082983
H	−3.404871	−2.093208	1.420785
C	−4.231992	−0.885594	−0.184660
H	−5.214453	−0.851264	0.304553
H	−4.410784	−0.989268	−1.265054
C	−1.645833	−2.063556	−1.540787
H	−0.712908	−1.655559	−1.951752
H	−1.584748	−3.161336	−1.565422
H	−2.459237	−1.759364	−2.214674
C	−3.643693	0.849479	1.550894
H	−2.982270	1.673839	1.849677
H	−4.682332	1.194853	1.651994
H	−3.491162	0.031401	2.269186
H	−2.106905	2.675478	0.346082
H	−0.058204	3.827975	0.462269
C	1.588287	−0.168832	0.003366
C	1.294645	−1.682124	−0.015246
H	0.327400	−2.086539	−0.250815
C	2.367345	−2.431426	0.204677
H	2.364987	−3.520137	0.208817
C	2.698481	−0.018053	−1.077165
H	2.551436	0.615848	−1.950101
C	3.769437	−0.764256	−0.844572
H	4.647623	−0.805977	−1.487605
C	2.282906	0.070719	1.373515
H	1.870589	0.776038	2.094296
C	3.358002	−0.681917	1.563219
H	3.973929	−0.665861	2.461664
C	3.656188	−1.635790	0.408709
H	4.536297	−2.260832	0.581398
C	2.274795	2.686214	−0.164282
H	3.011866	1.942953	0.137877
H	2.553243	3.112717	−1.138754
H	2.248371	3.499690	0.574495

PG-3-Me (47 atoms)

S_0 minimum

M06-2X/cc-pVDZ

$E(S_0) = -832.140278663$

C	0.269951	3.204711	0.276518
N	−0.862868	2.254869	0.329397
C	−0.510960	1.038511	−0.006812
C	0.928010	1.012166	−0.269434
C	1.303908	2.454372	−0.564791
H	0.612380	3.389115	1.307185
H	2.331838	2.698200	−0.276796
C	1.791718	0.004032	−0.003367
C	3.274608	0.093166	−0.333580
H	3.722166	0.981555	0.140193
C	1.522516	−1.295578	0.723883
H	0.548095	−1.740193	0.491431
C	2.695868	−2.184410	0.285895
H	2.860376	−3.026040	0.971346
H	2.491368	−2.602988	−0.712011
C	3.880532	−1.211833	0.223804
H	4.699101	−1.579057	−0.408655
H	4.296029	−1.049580	1.227810
C	1.567597	−1.007060	2.237204
H	0.774827	−0.303782	2.530454
H	1.426636	−1.943255	2.795272
H	2.529135	−0.568557	2.540798
C	3.462063	0.202974	−1.854728
H	2.967895	1.094268	−2.266417
H	4.531981	0.261219	−2.096367
H	3.044874	−0.680804	−2.361189
H	1.170349	2.696093	−1.630154
H	−0.079852	4.149025	−0.157988
C	−1.513330	−0.067466	−0.173984
C	−1.052067	−1.232125	−1.083389
H	−0.095383	−1.193245	−1.600687
C	−1.956486	−2.199452	−1.179699
H	−1.842747	−3.103432	−1.774950
C	−1.918419	−0.698549	1.177697
H	−1.482956	−0.356382	2.115815
C	−2.823060	−1.663235	1.044141
H	−3.243002	−2.237450	1.868597
C	−2.835944	0.401915	−0.841698
H	−2.946558	1.408163	−1.240739
C	−3.737753	−0.567308	−0.937994
H	−4.721031	−0.460034	−1.392522
C	−3.249654	−1.918233	−0.401451
H	−3.992278	−2.713291	−0.495392
C	−2.137120	2.722786	0.864350
H	−2.638709	3.372048	0.133621
H	−2.781013	1.877162	1.124708
H	−1.915308	3.310851	1.765452

PG-3-Me (47 atoms)
TS for propeller slippage
M06-2X/cc-pVDZ
$E(S_0)$ = −832.124328401
ω = 37.4037i

C	−0.135960	3.136984	−0.336653
N	0.920149	2.123313	−0.236906
C	0.493045	0.876816	−0.155911
C	−0.983193	0.877564	−0.186374
C	−1.412615	2.326377	−0.429498
H	0.064418	3.768611	−1.212501
H	−1.866384	2.412146	−1.426917
C	−1.955016	−0.070662	−0.048742
C	−3.411637	0.370821	0.065614
H	−3.640952	1.191447	−0.627576
C	−1.925610	−1.587520	−0.049552
H	−1.216679	−1.977551	0.687888
C	−3.360222	−2.014270	0.315341
H	−3.602467	−3.001336	−0.099476
H	−3.474959	−2.080718	1.406255
C	−4.228397	−0.889980	−0.248989
H	−5.230597	−0.851025	0.196988
H	−4.354671	−1.004688	−1.335193
C	−1.583454	−2.076120	−1.472669
H	−0.654331	−1.635560	−1.859070
H	−1.483660	−3.171038	−1.477798
H	−2.390653	−1.806766	−2.168561
C	−3.689091	0.837373	1.510107
H	−3.027170	1.653653	1.828905
H	−4.728575	1.184053	1.588082
H	−3.553194	0.008699	2.219105
H	−2.153391	2.671731	0.297593
H	−0.077286	3.771248	0.560080
C	1.581480	−0.173521	0.018382
C	1.295187	−1.688665	0.052340
H	0.321347	−2.110743	−0.118844
C	2.384834	−2.424720	0.243752
H	2.390973	−3.512241	0.280627
C	2.646678	−0.037092	−1.107945
H	2.459450	0.588532	−1.979921
C	3.733538	−0.770716	−0.900874
H	4.588757	−0.817403	−1.573005
C	2.324425	0.102544	1.356968
H	1.931389	0.825300	2.071699
C	3.415085	−0.636440	1.520956
H	4.066826	−0.595386	2.392268
C	3.675359	−1.615622	0.375573
H	4.565547	−2.229481	0.527215
C	2.273381	2.677687	−0.186746
H	3.020962	1.936365	0.094427
H	2.514125	3.099735	−1.172963
H	2.263225	3.494203	0.548538

PG-3-Ph (62 atoms)

S_0 minimum

CAM-B3LYP/cc-pVDZ

$E(S_0) = -1253.57748897$

C	1.157125	3.552778	0.757760
N	0.038877	2.630592	0.544139
C	0.385358	1.420308	0.185681
C	1.840868	1.371131	0.063338
C	2.278261	2.835942	0.007116
H	1.352140	3.637990	1.838587
H	3.258665	3.005456	0.464808
C	2.711835	0.326366	0.101858
C	4.185558	0.496595	−0.262011
H	4.659867	1.229476	0.411114
C	2.485203	−1.110271	0.513739
H	1.487443	−1.478662	0.256889
C	3.606533	−1.858871	−0.220370
H	3.792800	−2.851685	0.210576
H	3.333188	−2.004588	−1.276913
C	4.801882	−0.908941	−0.096775
H	5.578202	−1.098943	−0.849593
H	5.279827	−1.015267	0.886586
C	2.674170	−1.230118	2.040190
H	1.901051	−0.684581	2.590736
H	2.605149	−2.290225	2.324178
H	3.655282	−0.858543	2.367625
C	4.350109	0.990278	−1.708948
H	3.876133	1.963239	−1.890150
H	5.419403	1.093717	−1.940708
H	3.923897	0.267105	−2.420888
H	−0.934882	2.879847	0.700206
H	2.319076	3.201001	−1.030727
H	0.907893	4.545489	0.365279
C	−0.714878	0.403371	−0.007997
C	−0.467920	−0.722603	−1.037925
C	−1.465486	−1.706351	−0.998694
C	−1.007016	−0.227609	1.380676
C	−1.996037	−1.213895	1.342710
C	−2.090343	0.966608	−0.492451
C	−3.091078	−0.021045	−0.455033
C	−2.594337	−1.398805	−0.030033
H	−3.387115	−2.154341	−0.051613
C	0.492315	−0.764312	−2.041935
H	1.235112	0.024694	−2.143759
C	−1.440561	−2.774448	−1.882711
H	−2.220769	−3.536593	−1.839637
C	−0.436255	−2.847643	−2.848094
H	−0.414623	−3.682673	−3.549512
C	0.509798	−1.833797	−2.939818
H	1.268528	−1.864489	−3.723231
C	−2.391015	2.211150	−1.036468
H	−1.628924	2.974918	−1.191680
C	−4.380864	0.263378	−0.868237
H	−5.146981	−0.513262	−0.832094
C	−3.696334	2.495434	−1.454909
H	−3.920763	3.477029	−1.874051
C	−4.691630	1.536055	−1.353784
H	−5.707852	1.762823	−1.678258
C	−0.462456	0.153782	2.601515
H	0.283743	0.946102	2.671496
C	−2.400582	−1.854287	2.504274

H	−3.175703	−2.621617	2.462548
C	−0.872667	−0.486546	3.773246
H	−0.439314	−0.189065	4.729000
C	−1.827702	−1.494605	3.724469
H	−2.143729	−1.992676	4.641942

PG-3-Ph (62 atoms)
TS for propeller slippage
CAM-B3LYP/cc-pVDZ
$E(S_0) = -1253.54739715$
$\omega = 29.1072i$

C	−1.369886	−2.217537	2.466862
N	−0.250962	−1.454237	1.925583
C	−0.552326	−0.573956	1.002706
C	−2.005626	−0.701615	0.738458
C	−2.391213	−2.061329	1.346908
H	−1.702418	−1.762079	3.413042
H	−3.418806	−2.066068	1.724194
C	−2.988986	0.108395	0.256542
C	−4.341718	−0.450536	−0.180822
H	−4.928653	−0.644428	0.738686
C	−3.052269	1.623187	0.207384
H	−2.431928	1.971535	−0.630635
C	−4.521226	1.945570	−0.128929
H	−5.099932	2.045266	0.803732
H	−4.619314	2.891479	−0.677367
C	−4.996609	0.733953	−0.908375
H	−4.625167	0.771142	−1.946014
H	−6.089135	0.635096	−0.953103
C	−2.612879	2.275162	1.522598
H	−1.591398	2.005696	1.824559
H	−2.662776	3.371040	1.442440
H	−3.287509	1.969705	2.336091
C	−4.319240	−1.709897	−1.046649
H	−4.083195	−2.627574	−0.495594
H	−5.313122	−1.854489	−1.493925
H	−3.596941	−1.603890	−1.870491
H	0.717876	−1.622364	2.190782
H	−2.289636	−2.892702	0.634408
H	−1.058189	−3.250253	2.660790
C	0.700325	−0.072020	0.244233
C	0.835238	1.246004	−0.565851
C	2.046131	1.296868	−1.284511
C	1.950317	0.029920	1.177313
C	3.157577	0.086967	0.465663
C	0.981258	−1.225207	−0.787433
C	2.213482	−1.107422	−1.435944
C	2.980315	0.123772	−1.040435
H	3.931544	0.224929	−1.573951
C	0.040841	2.378897	−0.545912
H	−0.817300	2.435540	0.103232
C	2.373598	2.399277	−2.055077
H	3.315240	2.412722	−2.606946
C	1.516845	3.500417	−2.096534
H	1.771420	4.370122	−2.703399
C	0.369268	3.496380	−1.319803
H	−0.279700	4.372920	−1.293893
C	0.146215	−2.285873	−1.125866
H	−0.838626	−2.403202	−0.684213
C	2.623760	−2.041858	−2.375200
H	3.592678	−1.928263	−2.864667
C	0.552819	−3.224951	−2.075269
H	−0.114035	−4.049391	−2.330997
C	1.790848	−3.112129	−2.694074
H	2.105878	−3.850010	−3.432886
C	1.973292	0.222815	2.558203
H	1.054663	0.289395	3.142706

C	4.366491	0.224293	1.128440
H	5.296541	0.263563	0.558494
C	3.193281	0.369109	3.225417
H	3.197720	0.513591	4.306481
C	4.387324	0.345901	2.518706
H	5.337474	0.455287	3.042878

PG-3-Ph (62 atoms)
S_0 minimum
M06-2X/cc-pVDZ
$E(S_0) = -1253.79402686$

C	1.128931	3.596032	0.744951
N	−0.001184	2.684494	0.521311
C	0.347162	1.472743	0.171219
C	1.800558	1.417472	0.055032
C	2.242191	2.877988	−0.022716
H	1.328496	3.654610	1.826111
H	3.230853	3.047901	0.416766
C	2.647037	0.353238	0.109040
C	4.116047	0.477531	−0.285297
H	4.634034	1.193218	0.373702
C	2.391997	−1.066405	0.554349
H	1.369110	−1.413053	0.375256
C	3.450035	−1.857022	−0.228851
H	3.615856	−2.856382	0.194009
H	3.123407	−1.983310	−1.272844
C	4.687126	−0.952015	−0.153167
H	5.415731	−1.163050	−0.946440
H	5.207095	−1.085050	0.804233
C	2.685362	−1.146233	2.068507
H	1.971793	−0.554019	2.650358
H	2.602169	−2.194496	2.388755
H	3.698468	−0.795250	2.308371
C	4.236784	0.963329	−1.739454
H	3.768562	1.942025	−1.904484
H	5.297643	1.045914	−2.011443
H	3.769111	0.238420	−2.423667
H	−0.975045	2.931179	0.690977
H	2.255078	3.236776	−1.063248
H	0.886720	4.595239	0.368574
C	−0.721947	0.431786	−0.014818
C	−0.438578	−0.675097	−1.053500
C	−1.390591	−1.704027	−1.002698
C	−0.957935	−0.217342	1.373418
C	−1.905082	−1.246708	1.347537
C	−2.121000	0.941151	−0.471514
C	−3.080158	−0.088750	−0.425775
C	−2.520801	−1.449044	−0.017176
H	−3.281327	−2.237138	−0.031413
C	0.521152	−0.672943	−2.061130
H	1.223422	0.152291	−2.170844
C	−1.319067	−2.776895	−1.881184
H	−2.061677	−3.574528	−1.829590
C	−0.311993	−2.808222	−2.849034
H	−0.251944	−3.645521	−3.544282
C	0.585235	−1.749225	−2.952119
H	1.341059	−1.749422	−3.738353
C	−2.473674	2.175220	−1.011131
H	−1.741455	2.966085	−1.176734
C	−4.386896	0.144442	−0.823169
H	−5.119654	−0.662826	−0.781869
C	−3.796285	2.407675	−1.412225
H	−4.064865	3.379058	−1.827115
C	−4.752778	1.407864	−1.300180
H	−5.780135	1.595649	−1.611617
C	−0.402199	0.184963	2.583539
H	0.313543	1.007809	2.637822
C	−2.254995	−1.912617	2.514367

H	−2.996470	−2.712309	2.484197
C	−0.756915	−0.482924	3.760473
H	−0.315670	−0.173687	4.708101
C	−1.668014	−1.533795	3.724768
H	−1.939877	−2.050626	4.645156

PG-3-Ph (62 atoms)
TS for propeller slippage
M06-2X/cc-pVDZ
$E(S_0)$ = −1253.75970478
ω = 31.2411i

C	−1.387452	−2.200278	2.469256
N	−0.259812	−1.444176	1.929278
C	−0.554216	−0.565448	1.002830
C	−2.006974	−0.695289	0.728769
C	−2.401883	−2.050520	1.339069
H	−1.724406	−1.730322	3.405664
H	−3.432623	−2.043011	1.708374
C	−2.983404	0.120642	0.244254
C	−4.339144	−0.435282	−0.181423
H	−4.924597	−0.620611	0.740918
C	−3.033575	1.634062	0.184812
H	−2.440550	1.970196	−0.678501
C	−4.510722	1.956594	−0.116141
H	−5.071587	2.028720	0.829567
H	−4.622704	2.911300	−0.644570
C	−4.987780	0.752508	−0.910077
H	−4.603122	0.794293	−1.942410
H	−6.079398	0.655418	−0.961392
C	−2.553077	2.279472	1.489400
H	−1.542504	1.960137	1.783280
H	−2.552938	3.375533	1.401062
H	−3.236376	2.005515	2.306400
C	−4.308611	−1.698606	−1.041820
H	−4.114097	−2.616553	−0.475155
H	−5.286266	−1.823407	−1.527770
H	−3.549006	−1.605625	−1.832958
H	0.708016	−1.623493	2.196217
H	−2.298180	−2.888215	0.634525
H	−1.078422	−3.230606	2.673367
C	0.696818	−0.071426	0.243600
C	0.829273	1.223451	−0.599869
C	2.044313	1.260352	−1.315745
C	1.936112	0.061719	1.180245
C	3.149953	0.109044	0.477058
C	0.986900	−1.249631	−0.754663
C	2.224657	−1.145360	−1.398401
C	2.985895	0.100135	−1.032683
H	3.940249	0.191397	−1.562335
C	0.035466	2.358577	−0.601229
H	−0.814981	2.433549	0.056030
C	2.371685	2.344977	−2.113836
H	3.315542	2.347065	−2.661061
C	1.509227	3.442972	−2.185050
H	1.761625	4.297060	−2.813229
C	0.361774	3.457636	−1.405180
H	−0.286248	4.334370	−1.397410
C	0.152793	−2.319986	−1.070135
H	−0.835738	−2.424542	−0.631972
C	2.643427	−2.104504	−2.311349
H	3.615263	−2.001174	−2.796151
C	0.567743	−3.283102	−1.994021
H	−0.096422	−4.113440	−2.233745
C	1.811831	−3.184838	−2.607426
H	2.131921	−3.940953	−3.324171
C	1.937405	0.296191	2.555764
H	1.007955	0.364660	3.123960

C	4.351884	0.279894	1.148618
H	5.287023	0.312132	0.587599
C	3.150016	0.475467	3.231321
H	3.141991	0.652358	4.306744
C	4.353612	0.443626	2.536752
H	5.295589	0.579190	3.067976
